# Newborn Screening for Hemoglobinopathies and Thalassemias: Brief History, Recent Activities, and Global Status—2026

**DOI:** 10.3390/ijns12010008

**Published:** 2026-02-17

**Authors:** Bradford L. Therrell

**Affiliations:** 1 UT Health San Antonio, The University of Texas at San Antonio, San Antonio, TX 78249, USA; therrell@uthscsa.edu; Tel.: +1-512-921-1400; 2 US National Newborn Screening and Global Resource Center, Austin, TX 78759, USA

**Keywords:** newborn screening, hemoglobinopathies, thalassemias, dried blood spots, WHO, sickle cell disease

## Abstract

Newborn bloodspot screening (NBS) began in Guthrie’s laboratory in 1961 for phenylketonuria. A federal study the following year formed the basis for expanding NBS as a public health function. Diseases detectable through NBS gradually expanded, eventually including sickle cell anemia, which was included in the screening panel in New York in 1975. Universal inclusion of full population screening for sickle cell anemia was included in all US NBS programs by 2006. Through the years, NBS for sickle cell anemia has expanded to include other clinically significant hemoglobin disorders (both hemoglobinopathies and thalassemias). While NBS programs exist in most high-income countries, their implementation in low- and middle-income settings has been slow, with the inclusion of hemoglobin disorders occurring even more slowly. It is particularly noteworthy that the low-resource settings with the highest incidences of sickle cell diseases (Sub-Saharan Africa, the Caribbean Islands, and India) and therefore the greatest potential for benefitting from NBS, continue to struggle with its implementation. Recent advances in curative treatments further emphasize the importance of NBS in early disease identification. This report reviews some of the history of newborn screening for hemoglobinopathies and thalassemias and provides an update of related activities currently ongoing globally.

## 1. Introduction

### 1.1. Newborn Screening

Robert Guthrie, globally recognized as the ‘father’ of newborn bloodspot screening (NBS), described the beginning of newborn screening: “In the fall of 1961, I gave a talk about PKU [phenylketonuria] to the Association for Retarded Children in Jamestown, New York. By then, I was convinced that my test was going to be used to screen infants for PKU, so I devoted most of the talk to that subject. At that time, no one had yet begun newborn screening for PKU. A few days after the talk, I began to receive filter-paper specimens of blood from newborn infants in two Jamestown hospitals. Thus, screening had its start in Jamestown, New York, in 1961.” [1]. In 1962, building on Guthrie’s successes with NBS for PKU, the federal Children’s Bureau funded a national trial of his NBS technique with the goal of screening 400,000 newborns in 29 states [1,2,3].

State public health officials took note of NBS activities and their potential for reducing severe mental disability present with PKU, and by the end of 1963, the year in which Guthrie’s seminal publication on NBS for PKU was published [4], NBS mandates were in place in four U.S. states (Massachusetts, Oregon, Rhode Island, and Vermont). Since at the time, PKU patients were often housed in government institutions, cost savings from anticipated reductions in the number of institutionalized patients were recognized as a viable means for funding NBS. By the end of the decade, forty-five U.S. states had instituted mandates that all newborns should be screened for PKU [5].

### 1.2. Terminology

The language used in hemoglobin screening can be confusing. The nomenclature followed here is the same as that described/used in our earlier discussions of NBS for hemoglobin (Hb) disorders [6]. “Hemoglobinopathy” refers to hemoglobin structural variants such as HbS, HbC, etc., while “thalassemia” refers to decreased or absent production of globin chain(s). In either case, the essential ability of Hb to carry oxygen throughout the body can be negatively affected. Sickle cell disease (SCD) encompasses a “group” of disorders characterized by the presence of HbS, chronic anemia, painful related events, and associated complications. The homozygous presence of HbS (commonly referred to as sickle cell anemia) is denoted as SCD-SS (sickle cell disease-SS). Other prominent members of the SCD family, all of which were included in the original U.S. Recommended Uniform Screening Panel (RUSP) [7], include SCD-SC (Hb S,C disease or sickle cell disease-SC), and the sickle thalassemias, SCD-Sβ^0^thal (sickle cell disease-Sβ^0^thalassemia) and SCD-Sβ^+^thal (sickle cell disease-Sβ^+^thalassemia).

### 1.3. Sickle Cell Disease Newborn Screening—North America

Following Guthrie’s NBS publication in 1963, there was continuing research in his laboratory into screening procedures for other congenital conditions (usually asymptomatic at birth) [8]. Notable among these efforts was the 1973 report of an inexpensive and reliable NBS technique for detecting SCD-SS (and other hemoglobinopathies) at or near birth [9,10]. This procedure, a slight modification of the usual cellulose acetate/citrate agar electrophoretic procedures for adult blood, allowed for the detection of HbS despite the analytical complications due to high concentrations of HbF in newborns. The procedure cost about 0.03 USD per specimen for materials and allowed a technician to analyze up to 500 specimens daily [10]. The National Hemoglobinopathy Standardization Laboratory (NHSL) at the U.S. Centers for Disease Control [now the Centers for Disease Control and Prevention (CDC)] provided early guidance to improve screening interpretation [11]. Other early efforts at developing and improving NBS processes for hemoglobinopathy NBS at the time have been reviewed by Garrick [12].

Pilot studies of NBS for SCD began in other nearby populations known to have high incidences of SCD (like Puerto Rico [13] and Jamaica [14]) shortly after the NBS method was reported. Because of the ready availability of cord blood specimens (CBSs), they were sometimes used in early pilots [13,14,15], but their unsuitability for testing for many other NBS conditions limited this use (particularly when an expanded NBS panel was already operational or anticipated).

The introduction of automated dried bloodspot (DBS) punching equipment provided the impetus for NBS expansion during the 1970s [1,16]. The US National Sickle Cell Anemia Control Act (Public Law 92-294) in 1972 [17], the first federal program targeting a genetic disease [18], acknowledged SCD-SS (and ultimately other hemoglobinopathies) as a significant public health issue, and the NHSL was established at the CDC [6].

In 1974, the New York NBS law was amended to include screening for six additional conditions, including SCD-SS, and the New York NBS program became the first to require universal screening for SCD-SS (available in April 1975) [19]. Slowly, other state NBS programs began to add screening for SCD-SS, with only three states (New York, Colorado, and Texas) having implemented screening mandates by 1983 [20]. The dramatic results of the 1983 “Prophylactic Penicillin Study” (PROPS) demonstrated the efficacy of oral penicillin in preventing severe bacterial infections in children with SCD-SS [21] and led to a national consensus conference on “Newborn Screening for Sickle Cell Disease and Other Hemoglobinopathies” in 1987. The conference jury concluded that “every child should be screened for hemoglobinopathies … during infancy,” and that “state law should mandate the availability of these services.” Further, it encouraged the “centralization of laboratory services (for) efficiency.” [22].

Targeted federal funding for Hb NBS resulted in SCD inclusion in more than half of state NBS programs by 1990 [6]. While some programs initially opted to require targeted Hb NBS based on parents’ ancestry and some focused only on SCD-SS, a final push for full population (universal) Hb NBS led to universal Hb NBS mandates in all states by 2006 [see Table 1 in Ref. [6] for a complete listing of implementation dates in all states].

### 1.4. Sickle Cell Screening—Europe

Only slowly did other countries begin to add SCD to their NBS panels. In many jurisdictions (in the U.S. and elsewhere), NBS for SCD was targeted to “high-risk” groups based on race/ethnicity or geographic origin of ancestry. While NBS for PKU and certain other diseases like congenital hypothyroidism (CH) had expanded to locations outside of North America relatively quickly, this did not happen with NBS for SCD. 

One of the earlier European national NBS programs for SCD began in France [Guadeloupe (overseas France) in 1983/1984, Martinique in 1985, and pilots in Metropolitan France (Paris and Marseilles) in 1986—targeted to couples at risk [23].

In the 1970s in England, pockets of NBS for SCD had begun sporadically, which created an inequitable screening system nationally. Following a UK Health Technology Assessment review and economic analysis in 1999 [24], a National Health Service (NHS) plan to screen for SCD was developed, and SCD was formally added to the NBS panel in 2005 [25].

NBS for SCD also was implemented on a limited basis in a small number of other European countries: Netherlands (begun in 2007 and expanded to β-thal major and HbH-disease in 2017); Spain (Madrid region in 2003, Basque country in 2011, Valencia in 2012, and nationwide in 2015); Belgium (eight hospitals in Brussels in 1994, Liège Citadelle in 2002, and Liège Wallonia in 2007); Italy (several pilots since 2012); Germany (several pilots beginning in 2010); Ireland (pilots available since 2003), and Malta (available since 2017) [23].

In 2017, a Pan-European consensus conference on NBS for SCD in Europe assessed the current NBS for SCD situation and developed consensus-based recommendations. The fifty SCD experts representing 13 countries in Europe recommended universal NBS in all participating countries, initiation of case registries, data collection on clinical outcomes, development of shared comprehensive care protocols, increasing SCD public awareness, and focused education about SCD for all NBS stakeholders [26]. Since publication of the report, two countries (Germany and Portugal) have initiated national screening programs (discussed further in Section 3).

### 1.5. Sickle Cell Screening—Other Locations

Although known to have the highest incidences of SCD globally, both Sub-Saharan Africa (SSA) and the Caribbean islands had only limited screening pilots in the early days of NBS (1980s and 1990s) due to high poverty rates and untenable screening costs [27,28,29,30,31]. The current situation with respect to NBS for SCD in both of these locations, and in India (the third most prevalent location for SCD (but with no NBS pilots until around 2008 [32]) continues to be limited and will be more thoroughly addressed later.

### 1.6. Sickle Cell Screening—World Health Organization

The potential importance of NBS for SCD-SS was first documented in a report from the WHO Secretariat in 2006, which stated that, “There is evidence that the neonatal screening for sickle-cell anaemia, when linked to timely diagnostic testing, parental education and comprehensive care, markedly reduces morbidity and mortality from the disease in infancy and early childhood.” [33]. Further, this report noted that, “When health impact is measured by under-five mortality, sickle-cell anaemia contributes the equivalent of 5% of under-five deaths on the African continent, more than 9% of such deaths in West Africa, and up to 16% of under-five deaths in individual West African countries.” While World Health Assembly (WHA) resolutions in 2006 [34] and 2008 [35] recognized SCD-SS as a public health problem, there was no mention of NBS as a preventive measure. Acknowledging the extreme impact of SCD on health and well-being in SSA, the Africa office of WHO included NBS as one of its SCD strategies for the region in 2010 [36].

### 1.7. Sickle Cell Screening—Laboratory Testing

Over the years, technological advances have led to improved laboratory procedures for Hb NBS. Most Hb NBS programs now utilize either isoelectric focusing (IEF) or high-performance liquid chromatography (HPLC) for initial NBS laboratory testing, sometimes with a second-tier molecular test as part of the screening algorithm [20]. While capillary electrophoresis (CE) has been used for hemoglobin analysis since the early 1980s, its use in NBS has been limited. Commercial CE kits for NBS using DBS specimens have only recently become available and will likely be used by many screening laboratories in the future.

Next-generation sequencing (NGS) for SCD NBS can trace its roots to 1987, when McCabe (then at Baylor College of Medicine, Houston, TX) reported a technique for extracting DNA from DBSs using Texas DBS specimens [37]. Shortly afterwards, Guthrie sent a staff member to learn the extraction technique and collaborate in developing a molecular screening technique that could be used for Hb NBS [38]. In 1989, this technique was demonstrated to work using specimens from the Texas NBS program [39] and soon became a routine part of their Hb screening protocol [40]. Over time, Hb molecular analysis has evolved from basic DNA techniques to NGS that includes high-throughput simultaneous sequencing of millions of DNA fragments [41]. Pilot studies on the inclusion of NGS in NBS are ongoing, with continuing discussions of its evolution, predominantly in high-income countries.

### 1.8. Non-Sickling Hemoglobinopathy and Thalassemia Newborn Screening

The NBS laboratory technology for detecting SCD-SS and other SCDs can identify other clinically significant hemoglobinopathies and thalassemias (sometimes by choice and sometimes as an unintended consequence). Because newborns with beta thalassemias have a predominance of fetal hemoglobin (HbF), possibly another Hb, and little or no adult hemoglobin (HbA), they are usually identified through the differential diagnosis for other conditions (e.g., SCD-Sβ^0/+^thals are usually identified through the differential diagnosis of SCD-SS). Alpha thalassemias (α-thals), on the other hand, may be identified by the early presence and proportion of HbBarts, an abnormal hemoglobin composed of four gamma chains that forms when α-globin chains are deficient.

The increasing population diversity from immigration and globalization in the U.S. (and elsewhere) gradually expanded NBS for SCD to include other non-sickling hemoglobinopathies [42]. Because non-sickling disorders are found primarily in individuals of Mediterranean, Middle Eastern, and Asian ancestry, jurisdictions with these population groups were some of the first to implement Hb NBS, including California in the U.S. [43] and several European [23] and Middle Eastern [43,44] countries.

### 1.9. Review Limitations

In addition to providing a condensed history of NBS for Hb, this report reviews recent screening activities and summarizes the current status of Hb NBS globally, emphasizing the continuing challenges in implementing sustainable Hb NBS. While it is understood that NBS effectiveness is dependent on adequate medical services, the medical infrastructure and services available are beyond the scope of this report. Instead, the interested reader is referred to several references that have addressed these and other issues related to barriers to equitable sickle cell disease care globally [45,46,47,48,49].

## 2. Materials and Methods

Information reported here was accumulated from the published literature, other public sources, and professional contacts. These data were reviewed, consolidated, and summarized with the goal of providing the reader with a picture of current efforts focused on NBS for hemoglobinopathies and thalassemias internationally. In cases where available data were not clearly stated or appeared to be unreliable or in conflict with other data, an attempt was made to obtain new data or to resolve discrepancies through professional contacts, and any changes have been appropriately acknowledged. For verification and validation, commercial partners were sometimes contacted to clarify the available information.

A detailed 2024 report comprehensively reviewed NBS activities globally from 2020–2023 [31], including activities related to hemoglobinopathy and thalassemia NBS. Here, the focus will be on activities that have occurred since that time or other activities pertinent to understanding SCD NBS history. The organization and amount of information included for each section vary based on the amount of published information available.

## 3. Results

### 3.1. Screening Laboratory Procedures

Expansion and experiences in Hb NBS has led to continuing research into new analytical screening techniques in an effort to improve Hb screening quality and make it more affordable for low-resource countries. The 2019 standard from the Clinical Laboratory Standards Institute (CLSI) on Hb NBS provides a summary of these procedures and guidance for their use [41]. Included are acid and alkaline electrophoresis, IEF, HPLC, CE, tandem mass spectrometry (MS/MS), traditional molecular techniques, and NGS. 

A 2024 report from Europe (Italy) focused on screening methods for identifying SCDs and described several laboratory methods [50]. A 2025 report from the same group compared several analytical methods for quantifying HbS in DBS specimens and evaluated the stability of samples extracted and analyzed at different time intervals [51].

In the last two years, at least three reports from other European NBS programs have also described new Hb screening protocols. In Germany, a two-step procedure for detecting SCD in a high-throughput environment used quantitative PCR (qPCR) to pre-select relevant samples that are then differentiated by second-tier screening [52]. This preselection procedure was incorporated into a multiplexed qPCR assay for simultaneously detecting, in addition to SCD preselection, severe combined immunodeficiency disease (SCID) and spinal muscular atrophy (SMA). This initial screen was followed by a second-tier assay for SCD phenotype determination and carrier differentiation [53]. 

In France, a fully automated matrix-assisted laser desorption/ionization time-of-flight mass spectrometry (MALDI-TOF MS) platform was recently reported to compare favorably with screening by HPLC [54]. Also in France, in preparation for the expansion of the Hb NBS to full population (universal) screening, a dedicated MS/MS kit for SCD NBS was found to compare favorably to MALDI-TOF and cation-exchange HPLC procedures [55].

A recent research report from China describes a mass spectrometry-based, low-cost, high-throughput method for reliably screening newborns for thalassemia and abnormal Hb disorders [56]. With this first-line screening test, DBS specimen punches are digested with trypsin to release a series of Hb-specific peptides. Then, using HPLC and high-resolution mass spectrometry, disease-related peptides are incorporated into a prediction model using binary logistic regression to facilitate Hb screening. Reliable results were reported on specimens stored for up to one month.

A number of projects globally are considering molecular techniques for Hb detection. It has been used for many years as a second-tier in some screening programs, particularly in the U.S., but it is not yet routinely used as a primary screening test. A 2025 U.S. report in the U.S., notes that state NBS programs vary in their Hb NBS practices including primary screening tests, reporting procedures, follow-up protocols, access to genetic confirmation, and data management [57]. They argue that newborns diagnosed through protein-based screening alone may have diverse genotypes that alter the clinical expression of hemoglobinopathies. They further suggest that universal adoption of molecular testing for Hb NBS is needed to overcome diagnostic challenges. This can result in earlier treatment, which can be critical to the successful implementation of new therapies.

### 3.2. Point-of-Care Screening

To reduce costs and reduce the extensive follow-up of NBS testing results, there are many project reports concerning point-of-care (POC) screening tests. Early work was reported in summaries published in 2017 [58] and 2020 [59]. Since then, reports on other pilot activities have included studies in Angola [60,61], Democratic Republic of Congo (DRC) [62,63,64], Gambia [65], Ivory Coast [66], Mali [67,68], Namibia [69], Nigeria [70], and Tanzania [71]. Additionally, at least three POC studies have also been reported in India [72,73,74,75] along with one in Haiti [76]. Illustrating the current popularity of the HemoTypeSC™ POC kit, which uses whole blood rather than DBSs, a 2024 report outlined a qualitative study to be undertaken in seven countries (Ghana, Mali, Nigeria, Tanzania, Uganda, Zambia, and Zimbabwe) that will explore some of the factors that may ultimately influence the adoption and sustainability of this test in primary healthcare centers. This project is part of a larger SickleInAfrica (see description below) NBS project on “Clinical and Implementation Research for Newborn Screening for Sickle Cell Disease Using Dried Blood Spots for Point-of-Care Tests” [77].

### 3.3. Carriers

In addition to the laboratory carrier detection/cutoff issues when screening for α-thalassemias, detection and follow-up for sickle carriers [sickle cell trait (SCT)] have also been the subject of much discussion over the years (even until now), both in the U.S and in other countries [78,79,80,81,82]. While Hb carriers are usually reported as part of U.S. NBS protocols, the degree to which there is active follow-up has always varied across programs, particularly since identification of carriers is not the goal of most NBS efforts [83,84]. At least one program (Texas) has formally included SCT on its screening panel. A recent US report used SCT result reporting as a use case for NBS program participatory workflow analysis to document program diversity, opportunities, and challenges. The study is intended to improve patient education and follow-up with future work planned to broaden the analysis to include parents, caregivers, and other stakeholders [85].

While SCT is generally considered to be a benign condition, adverse events have been reported infrequently [86]. In 2010, the U.S. National Collegiate Athletic Association (NCAA) began requiring Division I student-athletes to know their SCT status as part of a legal settlement resulting from the death of a football player who died from causes attributed to SCT [87]. The requirement now extends to Division I, II, and III student athletes. This policy remains medically controversial, is possibly stigmatizing, and continues to be opposed by the hematology community and professional organizations [88]. The SCT results from NBS are sometimes used to satisfy the requirement [89]. The California NBS program recently analyzed mortality data for 94,240 SCT-positive screens from 1991–2013, determining that further population-level research into causes of death is needed to inform clinical management and counseling for SCT [90].

### 3.4. Alpha-Thalassemias

The U.S. RUSP received formal approval from the Secretary of Health and Human Services (HSS) in 2008 and currently guides NBS in the U.S. [7]. Included are SCD-SS, SCD-SC, and SCD-Sβ^0/+^thal as “core” conditions and all other hemoglobinopathies as “secondary targets”. In 2010, HbH disease, an α-thal more prevalent among persons of Mediterranean, Middle Eastern, and Asian descent, was nominated for inclusion as a core condition, but its inclusion was not approved [91]. Despite not being included as a RUSP core condition, HbH-disease is still detected in most U.S. NBS systems [92] since its biochemical marker, HbBarts, is detectable by Hb NBS laboratory techniques [93]. Its frequency among various racial/ethnic groups in California has been reported [94], and these data were used in lieu of pilot testing data to support the addition of Hb to the Philippine NBS panel beginning in 2014 [95]. As a result, HbH-disease was confirmed in 2427 newborns from 2014 through 2023 [96]. Elsewhere in Asia, HbH-disease NBS has been limited to pilot projects in some parts of Thailand (CBS prevalence studies) [97,98] and China [99]. An improved mass spectrometry-based laboratory procedure for thalassemia detection has also been reported in China (see discussion in Section 3.1) [56]. Similarly, in the Middle East, NBS projects have been reported in Bahrain [100], Lebanon [101], Oman [102], Saudi Arabia [103], and the United Arab Emirates [104], among others, usually preliminary to implementing premarital or prenatal screening programs.

In order to optimize the NBS algorithm and minimize unnecessary follow-up and detection of carriers (not a usual goal of NBS), laboratory-specific cutoffs for HbBarts must be developed as part of the HbH-disease screening protocol. HbH-disease occurs when three of the four alpha globin genes are deleted or functionally impaired. Because HbBarts concentrations vary with the newborn’s age, and there is some degradation of hemoglobin in DBS specimens over time, determining an optimum cutoff can be challenging. As examples, three separate reports of quantitative cutoff concentrations for HbH-disease based on the percentage of HbBarts are referenced: HPLC screening in California (25%) [105], HPLC screening in the Netherlands (22.5%) [106,107], and CE screening in China (10%) [108]. Because the presence of Hb Barts may not be aggressively followed on results outside of the expected range, a recent two-year mixed methods study at Cincinnati Children’s Hospital in Ohio looked at the clinical utility of adding genetic testing to standard procedures for possible alpha-thalassemia trait and also the related educational needs of primary care providers [109]. The addition of genetic testing to the NBS protocol for possible alpha-thalassemia trait had very high clinical utility. Education and genetic counseling were not recommended without subsequent specific genetic verification.

### 3.5. Screening in Sub-Saharan Africa

In his 2025 review [110], Obeagu notes that, “In Sub-Saharan Africa, the prevalence of thalassemia, particularly beta-thalassemia, is significant due to genetic predisposition and historical factors such as malaria endemicity.” And, “Early diagnosis, effective management, and prevention strategies are limited by factors such as poor health care infrastructure, a lack of skilled professionals, and insufficient access to necessary medical treatments.” In addition to thalassemias, approximately 75% of the global burden of SCD is also present in SSA. A 2025 report estimated the pooled prevalence of SCD-SS in African populations and examined the regional, demographic, and temporal variations from 1994 to 2024 [111]. This study demonstrated substantial and geographically variable public health challenges across Africa and emphasized the need for region-specific interventions, expanded NBS programs, improved diagnostic accessibility with quality assurance for point-of-care technologies, and continued surveillance to address geographic gaps. 

A 2019 meeting of 150 representatives from 20 countries, including 11 from SSA, sought to develop meaningful collaborations for improving child health with a focus on NBS for SCD. The meeting report describes the screening activities of the African countries that attended [112]. In combination with the country-by-country report of activities worldwide published in 2024 [31], the interested reader is provided with an overview of the challenges in implementing SCD NBS in these low-resource settings. Several ongoing efforts that can have an effect on NBS implementation exist, and a 2025 workshop as a side event of the 5th Global Congress on SCD focused on the current screening situation in Africa, possibilities for the future, and strategies to continue moving forward [113].

The SickleInAfrica consortium of clinicians, academics, and scientists from existing SCD programs in SSA facilitates SCD research and its subsequent translation into healthcare and health outcomes [114]. It has three initiatives: Sickle Pan-African Research Consortium (SPARCO) to develop SCD research capacity through a multidimensional approach (sites in Tanzania, Nigeria, and Ghana), Sickle Africa Data Coordinating Center (SADaCC) to coordinate data standardization and communications for SPARCO (in South Africa), and Sickle Cell Pan African Network (SPAN), a network of researchers, clinicians, funders, and centers working in SCD in 22 sites in 17 African countries. “To implement standardized newborn hemoglobinopathy screening and early intervention for children with SCD in SSA,” the American Society of Hematology (ASH) has instituted the Consortium on Newborn Screening in Africa (CONSA), consisting of seven countries—Ghana, Kenya, Nigeria, Liberia, Tanzania, Uganda, and Zambia [115]. Other activities exist, including a consortium of collaborators from six SSA countries (Angola, DRC, Ghana, Liberia, Nigeria, and Tanzania) who issued a 2022 report that systematically assessed the enablers and barriers to implementing NBS programs for SCD in these six countries. Their report concluded that while NBS for SCD is feasible, sustainability is an issue and, therefore, government partnerships and funding should be a priority as part of program development [116].

As NBS has led to earlier diagnosis of SCD, treatment availability has emerged as a pressing challenge. The historical, clinical, and public health perspective of SCD in SSA children were reviewed in a 2022 report [117] and a 2024 article “examines the policy framework for NBS in SSA; the methods, processes, barriers, and enablers of NBS; and enrollment in comprehensive care to make available the evidence-based interventions that caregivers need to access in order to save the lives of babies with SCD” [118]. Interestingly, public-private partnerships have played a role in SCD treatment and, hence, in NBS implementation. Novartis, a global medicines company, and the Sickle Cell Foundation of Ghana (SCFG), along with other partners, worked with the government to include hydroxyurea, a disease-modifying generic treatment for SCD, into its National Health Insurance Scheme in 2022 [119]. Other private companies, most notably Revvity (formerly PerkinElmer, a science-based solutions company active globally in NBS) has collaborated with Novartis to facilitate the introduction of NBS for SCD throughout SSA [120]. A 2022 report notes the ongoing efforts of the Norvartis Africa Sickle Cell Disease program to expand the eleven SCD treatment centers in Ghana to Kenya, Uganda, and Tanzania, while discussing the possibilities for using the U.S. President’s Emergency Plan for AIDS Relief (PEPFAR) to bring support therapies for SCD to SSA [121]. At least two recent reports discuss the lack of treatment availability, defining it as a “public health imperative” and suggesting strategies for expanding and appropriately using treatments like hydroxyurea [122,123].

More recently (2024), reports have focused on the evolving SCD treatments and their inequitable distribution. One report discusses the eventuality of somatic gene therapy for SCD in SSA, suggesting that it provides the opportunity for stakeholders to work together now to develop education materials, decision-making toolkits, and patient-centric governance for gene therapy. This includes the creation of harmonized ethical and regulatory frameworks for gene therapy [124]. The other report notes that curative hematopoietic stem cell transplantation (HSCT) is available in only 7 African countries, in Africa, and the few patients who can afford HSCT and who have suitable sibling donors usually must travel out of the country for treatment and return home, where expertise and resources for post-transplant follow-up are lacking [125]. Another 2024 report discusses the need for ambitious action to ensure that SCD treatments and cures are affordable and accessible in SSA, where the majority of cases exist, and the biggest impact can be felt [126].

Noting that 80–90% of children with SCD die before their fifth birthday, recent reports from Uganda, Cameroon, and Nigeria discuss the possibilities for improving SCD outcomes by integrating SCD screening and care into well-established primary healthcare programs and services like vaccination, antenatal care, and non-communicable disease clinics [127,128,129]. Genetic counseling before and after SCD NBS is also a part of screening in Kano, Nigeria [130], and a genetic counseling training program connected to the SCD NBS program has existed in Ghana for over 10 years [131]. Also in Ghana, reports in 2023 and 2024 investigated the financial impact on parents caring for children with SCD (2% of the population with SCD and 25% with SCT). Both reports noted families’ financial stresses, the lack of understanding of the difficulties by policy makers, and the need for increased financial support through subsidized medications and laboratory services, and counselling and psychological support services [132,133].

Other reports have focused on showing the value of NBS in jurisdictions lacking reliable incidence data or updating available data based on NBS results. A study report in São Tomé and Príncipe in 2024 was the first of its kind to estimate the prevalence of SCT in women of reproductive age (13.45%), supporting the need for SCD NBS [134]. A project in Cape Verde on a small population (346 newborns, 6% carriers of HbS) successfully demonstrated that HPLC and molecular analysis could be used to implement NBS for SCD there [135]. A report from Angola described the first year (2023–2024) of a public-private partnership project (with Revvity) in a large hospital in Luanda (in collaboration with the NBS program in Lisbon, Portugal), to develop Hb prevalence data (HPLC screening, 9074 newborns, SCD-SS = 1:75) [136]. Part of this project also involved training technicians in laboratory operations in Portugal for return to develop laboratory capabilities in both Angola and Nigeria. Three reports updated Hb prevalence data from DRC in Kisangani (HemotypeSC^™^ screening, 1432 newborns, SCD-SS = 1:46) [137], Lubumbashi (CE screening, 588 newborns, SCD-SS = 1:22) [138], and Bukavu (HemotypeSC^™^ screening, 4496 newborns, SCD-SS = 0.22%, SCT = 8.16% but follow-up was inefficient to determine true prevalence) [139]. These data add to DRC prevalence information already referenced for Kindu [63] and Butembo/Beni [64]. A collaboration between U.S. researchers and the Malawi HIV Early Infant Diagnosis program used leftover HIV DBS specimens to establish an incidence of SCD and other hemoglobinopathies in Malawi (10,529 newborns, SCD = 1:752) [140].

As NBS spreads in SSA, policies have emerged that encourage screening; however, limited data have been published regarding screening acceptability among parents or other stakeholders. At least three reports have recently emerged addressing parental participation in the NBS process. A recent report from Kenya, where a 2020 policy on NBS was enacted in level 2–6 healthcare facilities, assessed factors associated with the acceptability of NBS among mothers of newborns at a county teaching and referral hospital in western Kenya. Ninety-four percent of mothers accepted NBS for SCD, suggesting that the screening policy should be expanded to all birthing facilities [141]. A 2024 report reviewed a project in Nigeria that assessed the effect of education on the knowledge about SCD and NBS among pregnant women using a community-based framework. Health education was associated with significant improvement in SCD and NBS knowledge [142]. Another report from Nigeria in 2025 assessed the awareness, attitude, and acceptance of NBS for SCD among health workers and caregivers at immunization and antenatal clinics in selected Primary Healthcare Centers [143]. There was a high level of awareness of NBS for SCD among health workers. Caregivers had a low level of awareness but a high level of acceptance. Among the groups studied, education was the major factor determining both knowledge and attitude. A report from Ghana explored the views of community members and healthcare workers about the role of counselling to reduce parental fears and anxiety about newborn screening for SCD in rural Northern Ghana [144]. While counselling was planned as a key component of the SCD NBS program, training to improve knowledge and counselling skills, and a positive attitude towards parents was recommended to improve counselling services.

Most recently, a 2026 report from Nigeria documents the activities of a three-day national NBS workshop collaboratively organized by the Newborn Screening Consortium–Nigeria (NSC-N), the Federal Ministry of Health, Nigeria, Revvity, and other international partners to review congenital disorders that may be suitable for NBS, implementation barriers, and national NBS needs [145]. The NBS 3-day workshop developed a plan to initiate and integrate NBS for SCD and congenital hypothyroidism into the Nigerian healthcare system, including integration into routine maternal and child health services, establishing a national screening database, and developing a robust legislative and policy framework. Another 2026 report (previously referenced) reviews the continuing gaps in addressing NBS for SCD in SSA, noting in particular that planning and funding remain as significant challenges, and the lack of global NBS standards continues to perpetuate the screening inequities worldwide [49].

### 3.6. Screening in India

Most babies with SCD are born in the DRC, Nigeria, and India [146]. Newborn and population screening has progressed in India, and comprehensive care, pneumococcal prophylaxis, and disease-modifying therapy with hydroxyurea are all now freely available in government-run clinics. In 2013, SCD screening was included in India’s national child healthcare program, Rashtriya Bal Swasthya Karyakram (RBSK), and its inclusion provided greater opportunities for the addition of SCD to state NBS programs [147]. Despite these efforts and numerous pilot studies demonstrating its value, there is still no national NBS program, and screening at the state level is limited. A 2018 review summarized the results of various screening pilots and concluded that NBS was feasible, even in the higher prevalence tribal regions, and noted the need to follow 2016 guidelines promulgated by the Ministry of Health and Family Welfare [148].

In 2023, the Government of India launched the “National Sickle Cell Anemia Elimination Mission” (NSCAEM), a 3-way partnership with for-profit and nonprofit institutions, industry, and academia. Significant funding has been made available for screening, treating, counseling, educating, and developing technologies, novel therapies, and gene therapies. The ultimate goal is to eliminate SCD as a public health problem by 2047 [149,150]. An online publicly available information dashboard provides up-to-date cumulative and daily data on population screening progress (without differentiating NBS) [151]. While this project seemingly opens the door to increased NBS, no data exist that indicate progress in this regard, although recently added PowerPoint presentations on the main Mission website include NBS [152].

A 2025 report reviewed the success of an SCD NBS project at a Centre of Excellence for SCD (COESCD) in Rajasthan, showing the presence of abnormal hemoglobins in 1.87% of the 26,642 neonates screened, further demonstrating the potential value of NBS [153]. Another 2025 report described the results of a study of screening, follow-up, and comprehensive care for SCD babies in seven SCD-prevalent tribal regions seeking to identify barriers and facilitators to NBS implementation [154]. The findings suggest the need for a holistic approach addressing socio-cultural dimensions to effectively integrate NBS into the health system and provide insights for shaping NBS policies in SCD-prevalent areas.

Despite the fact that NBS with DBSs is the predominant mechanism for screening newborns globally, there are still ongoing debates over the specimen of choice among some policymakers in India. Thus, pilot projects continue with a goal of demonstrating the reliability and usability of the DBS specimen. A 2025 report illustrated comparability between CBS and DBS specimens and demonstrated the value of using DBS specimens for easier transport in remote rural areas (HPLC Hb NBS methodology) [155]. Another recent report described a CBS protocol for a multicentric cohort study on SCD neonatal screening in six Indian States with a high prevalence of SCD (Rajasthan, Odisha, Tamil Nadu, Maharashtra, Madhya Pradesh, and Gujarat) to pave the way for screening implementation [156].

### 3.7. Screening in Other Jurisdictions

#### 3.7.1. North America

The worldwide reach of Hb NBS is illustrated in the regional tables published in 2024 [31]. Since that time, several updates have been reported. For North America, reports of activities both in the U.S. and Canada have appeared.

A report on Hb NBS over the last two decades in Iowa (U.S.) documented increased sickle and non-sickle case detections and the importance of program evolution to meet the healthcare needs of new and underserved minority populations [157].

A recent data project at the Centers for Disease Control and Prevention (CDC) provided data for SCD prevalence comparisons in eleven states participating in the project [158]. This project accumulated case detection data from 2016 through 2020, which allows for comparisons with previously reported 20-year data (1991 through 2010) from fifty-one U.S. programs [159]. As part of the CDC study, data from Colorado (U.S.) The Sickle Cell Data Program (created in 2021 to enhance surveillance and public health efforts for SCD) were used to identify comparative baseline prevalence estimates for SCD in Colorado [160].

The Arkansas Hb NBS program reviewed long-term follow-up data in Arkansas from 2011 through 2023 and identified gaps in follow-up care, namely low utilization of available services and distance of specialty care [161].

In Canada, data reported from Hb NBS in Quebec (Canada) since its implementation in 2013 confirmed the positive impact of universal vs. targeted NBS for SCD in the province, suggesting the importance of its adoption in other Canadian provinces (currently, universal Hb NBS is only available in seven provinces and two territories out of thirteen) [162]. A 2026 report from Alberta notes the first reported detection of Hb Chapel Hill in a newborn. This Hb mimics HbS on standard screening procedures (both HPLC and CE), emphasizing the potential importance of molecular testing as a second-tier screening test [163].

#### 3.7.2. Europe

Since late 2023, a number of reports have addressed new Hb NBS activities in Europe. In the UK, a guideline for screening and diagnosis of significant hemoglobinopathies from the British Society for Haematology was published in 2023 [164]. While guidance for SCD NBS was left to the National Health Service Sickle Cell and Thalassaemia Screening Programme, it recommended that all babies under 1 yr of age arriving in the UK should be screened for SCD.

In Spain, a 2024 10-year review on 1531 patients with SCD or thalassemia (37% detected by NBS) in the Spanish nationwide hemoglobinopathies and rare anemias registry was published [165]. Four additional reports reviewed information from Spanish autonomous regional screening programs: Murcia–data since the program began in 2016 (CE screening 104,083 newborns, SCD = 1:10,000) [166]; Catalonia–data from 2015 to 2022 (CE screening 506,996 newborns, SCD = 1:3169) [167]; Western Andalusia–data from 2018 to 2021 (HPLC screening 111,205 newborns, SCD = 1:12,356) [168]; and Madrid–data from 2003 to 2018 on a cohort of 187 SCD patients diagnosed from NBS (HPLC screening 1,048,222 newborns, SCD = 1:5552) [169].

NBS for SCD was officially added to the German NBS panel in October of 2021. A review of SCD NBS data from October 2021 through May 2024 (multiplex PCR) found a SCD prevalence of 1:5300 (410,273 screened) [170].

Three reports from Italy discuss regional SCD NBS: a 10-year review of the first regional experience in Italy in Friuli-Venezia Giulia–data from 2010 to 2019 (HPLC screening 11,956 newborns, SCD = 1:249) [171]; and a review of the essential NBS system components, including a costing analysis framework, for implementing NBS SCD in Modena [50,51].

A Letter to the Editor from the Netherlands reviewed the universal SCD NBS program accomplishments from 2007 to 2023, which included the detection of 540 cases of SCD and showed improved survival rates with the highest morbidity in the first four years of life [172].

Data from two phases of a pilot study in Portugal from May 2021 to January 2022 supported the addition of SCD to the screening panel: data from the Lisbon/Setubal region (24,130 newborns, SCD = 1:928) and nationally (164,087 newborns, SCD = 1:2449) [173]. SCD was added to the Portuguese NBS panel in January 2024.

The national French SCD NBS program, which has been targeted to high-risk individuals since its beginning, became universal on 1 November 2024 [174].

#### 3.7.3. Asia Pacific (Excluding India)

Aside from reports already noted from India, China, and the Philippines, Hb NBS is not yet a priority in Asia and the Asia Pacific. Several studies of various aspects of Hb NBS in China (but not previously referenced) have recently been reported. A 2024 report described a three-tiered thalassemia prevention and control strategy in China that includes carrier screening, prenatal diagnosis, and NBS in provinces with high rates of thalassemia. This study of 2000 newborns demonstrated that combining CE, hotspot DNA testing (with a locally produced kit), and third-generation sequencing for a comprehensive analysis of thalassemia alleles (CATSA) was extremely effective in identifying thalassemia variants as part of the thalassemia prevention and control strategy [175]. A second report reviewed and analyzed NBS data from Beiliu Maternity and Child Health Hospital (Guangxi Zhuang Autonomous Region) from January to December 2023 (CE screening, 4134 newborns, variable screening results listed) [176]. Noting the relatively high prevalence of thalassemia in China, another recent report reviewed the current research on thalassemia screening and the latest research on the treatment of thalassemia utilizing gene therapy [177].

#### 3.7.4. Latin America and the Caribbean

NBS for SCD in Latin America (LATAM) and the Caribbean dates to 1974, when the first program in the region was initiated in Jamaica [14]. The SCD NBS situation in the Caribbean was recently reviewed [30]. Use of CBSs for SCD occurs in Martinique, Jamaica, and St Lucia, while heelstick DBS specimens are used in Guadeloupe, Tobago, Grenada, French Guiana, and a pilot project in Saint Lucia. SCD NBS specimens from French and Dutch territories are sent to screening laboratories in the respective countries in Europe.

Two recent reports reviewed SCD NBS activities in South American mainland countries considered as part of the Caribbean region: a pilot screening program for SCD in Suriname (CE screening, 5190 newborns, SCD = 1:157) was evaluated using an evidence-based framework, and its implementation was recommended [178]; and a review of thirty years of data from the French Guiana SCD NBS from 1992 to 2021 (175,593 newborns, SCD = 1:213) confirmed the highest SCD prevalence among French territories [179].

Capacity building and networking to make newborn screening for sickle cell disease a reality in Haiti were reported in 2018 [180], and a 2024 report describes implementation of the Haiti Comparative Study of Children in Haiti and Miami with Sickle Cell Disease (CSHSCD). This hospital-based SCD NBS project exists in four locations and demonstrated that implementation of NBS and SCD programs in Haiti was feasible (from May 2021 to May 2022, 8224 screened, SCD = 1:135) [181].

An audit of a pilot SCD NBS project in Antigua and Barbuda, a twin island state, over the period September 2020 through December 2021 (laboratory testing in Guadeloupe) provided prevalence data that supported the value of SCD NBS (1550 screened, SCD = 1:222) [182].

In LATAM, SCD was added to the ongoing NBS program in Minas Gerais (Brazil) in 1998. As part of the Brazilian government’s centralization of NBS in 2001, SCD was included in the phased national NBS implementation, becoming national in 2013 [183]. Full population Hb NBS programs in LATAM were also implemented in Costa Rico and Uruguay [31].

There have been at least four recent reports on SCD NBS activities in the region, with at least two from Brazil: an overview of the progression of SCD in the past two decades discussed equity issues and noted that only 14 of 26 states have implemented screening since the 2001 signing of the ordinance adding SCD to the national NBS program [184]; and an evaluation of SCD NBS in the state of Mato Grosso from 2005 to 2019 (599,534 newborns, SCD = 1:3013) [185].

A 2022 report suggested the inclusion of hemoglobinopathies in the ongoing efforts to implement a broader NBS program in Guatemala (HPLC, 3006 newborns, HbS allele 1:137) [186], and a 2024 report evaluated the data from the first SCD NBS program in Colombia, Prevención y Genética (PREGEN) established in Bogotá in 1988, from 2006 to 2019 (HPLC screening, 40,183 newborns, SCD = 1:207) [187].

#### 3.7.5. Middle East and North Africa

While there are some countries in the region that include SCD as part of their NBS programs, there is no consensus on its inclusion. The rate of consanguinity is high, and as an alternative to NBS, there are many premarital and prenatal screening programs for SCD in the region [31]. Recently, there have been at least three reports of SCD NBS activities in the region.

A 2024 report from Tunisia reviewed a small pilot project in one hospital in central Tunisia seeking to gain insight into whether there are hemoglobinopathies in the population detectable through NBS (156 newborns, 12% β-thal, 0.3% α-thal, molecular confirmation) [188]. More recently, a CBS screening study in Northern Tunisia (Bizerte region) used CE to identify several clinically significant thalassemias and hemoglobinopathies, highlighting the feasibility and reliability of screening and the need for broader implementation across the country [189].

In Morocco, a study from 2016 to 2023 evaluated the feasibility of integrating NBS into diagnostic laboratories as a model for possible expansion nationwide. Six conditions were considered, including Hb NBS (IEF screening, 5411 newborns, only carriers detected 0.9%, including carriers for HbC, HbS, and HbBarts) [190].

A brief report from Saudi Arabia noted that the Saudi Ministry of Health’s Blood Disorder Administration added SCD and G6PD to the current mandatory national NBS panel in April 2023 and reported data from a quantitative study from April 2023 to June 2024 (HPLC screening, 331,151 newborns, SCD-SS = 1:622) [191].

### 3.8. World Health Organization

In 2024, as a means of meeting United Nations 2030 Sustainable Development 3.2 for good health and well-being (end preventable deaths of newborns and children under 5) [192], WHA encouraged WHO member states to “consider implementing a universal newborn screening programme including comprehensive birth defect screening, including specific needs and considerations for diagnosis, management and long-term care of children with birth defects” [193]. In support of the WHA resolution, the WHO is currently developing a suggested broad-based NBS framework to support low-resource countries in selecting initial screening conditions and integrating screening into local public health systems [194].

## 4. Discussion

It is easy to see where the action is in NBS activities related to Hb detection from the disproportionate number of recent publications related to NBS for SCD in SSA. The large amount of work ongoing there is critical to advancing NBS for SCD, as it is in India, the Caribbean, and other high-incidence locations like Brazil. As important is the work ongoing to improve laboratory techniques and move towards molecular testing strategies to allow for earlier diagnosis and treatment [109,195].

As curative treatments for SCD evolve, early detection strategies like NBS become increasingly important, along with their efficient administration and seamless inclusion into public health prevention strategies. The initiation of NBS for SCD-SS (and eventually for other hemoglobinopathies) is now 50 years old. It has been almost 40 years since a U.S. national consensus development conference noted that the benefits of screening were so compelling that universal SCD screening should be provided in every state (finally coming to fruition in 2006). Still, a 2026 publication notes that, “In the United States, fragmentation of policies by state, lack of federal guidelines, and quality assurance practices continue to hinder more effective adoption of NBS.” [196]. Provider education gaps, including urgency (like penicillin prophylaxis) and barriers for families (insurance, transport, language), have led to missed specialist appointments and fragmented care, despite NBS’s success in improving survival. Identifying and bridging existing gaps can improve health outcomes and reduce the global burden of SCD.

Challenges to sustainable NBS for SCD exist worldwide. In addition to the U.S., there are still opportunities in Europe to comply with the recommendations of the 2017 Pan-European consensus conference on NBS for SCD, which included, “universal NBS in all countries participating in the conference, … registries and … shared clinical protocols … raising public awareness … focused education…” [26]. In Brazil, despite a national Ministry of Health NBS program for SCD initiated in 2013, it is still not available in some states [183]. There, the main challenge seems to be organizing the public health network to provide comprehensive care for patients diagnosed through NBS. While considerable gains have been achieved, including the development of a Brazilian SCD registry, there continues to be a need for increased public education and health promotion about the disease to continue to reduce mortality and enhance patients’ quality of life.

As one might expect, Implementation of SCD NBS has been particularly problematic in the poorer, high-incidence countries in SSA, the Caribbean, and India. In SSA, where approximately 75% of the 500,000 newborns with SCD worldwide are born, NBS has not developed, primarily as a result of poor economic conditions and low resources [197]. NBS for SCD combined with administration of prophylactic interventions has been shown to be cost-effective in the majority of SSA countries [198] and could be a major contributor to reducing SCD-related mortality [199]. The four most common barriers to SCD NBS in SSA have been identified as governance (ownership and role of government), technical operations (testing, tracking, and follow-up), cultural influences (knowledge and beliefs), and finances (role of government and insurance) [116]. A recent review of sustainability strategies noted that development and implementation of less expensive and reliable POC screening tests may be a game changer [197]. To this end, completion of the SickleInAfrica multi-national collaborative project on the use of DBS specimens in POC testing may hold the key [77].

Indeed, home-grown projects and collaborative initiatives in Africa are beginning to make a difference. The public-private partnership in Ghana that has resulted in coverage of SCD treatment by national insurance provides a useful model for other SSA countries [119]. This is also true of the partnership in Malawi between local healthcare programs and developed screening programs in other countries [140]. Other creative initiatives worth noting include SCD NBS integration into immunization activities in Cameroon [128] and Nigeria [129], development/inclusion of genetic and sickle cell counseling in SCD NBS in Nigeria [130] and Ghana [131], development of large-scale community-based educational programs on SCD NBS focused on pregnant women in Nigeria [142], and development/sharing of the SCD NBS follow-up smartphone app in Ghana and CONSA countries [31].

In India, the NSCAEM government initiative seeking to eliminate SCD by 2047 [131] and inclusion of SCD in the RBSK program to expand NBS [130] appear to provide hope for broader SCD NBS implementation. Likewise, the Caribbean Network of Researchers on Sickle Cell Disease and Thalassemia (CAREST), formed to advance SCD activities in the Caribbean [200], continues to work with developing programs such as Antigua and Barbuda [166] to coordinate pilots and laboratory testing. Coupled with activities from other established screening programs, these efforts appear to be helping to expand SCD NBS in the region. And, demonstrating the long-term value of NBS, recent updates on the 311 SCD-SS cases detected by NBS between 1973 and 1981 (Jamaica Sickle Cell Cohort Study) continue to contribute to the natural history of SCD [201,202].

An unintended consequence of NBS for SCD has been the detection of thalassemias. Thalassemia detection did not receive the focus given to SCD primarily due to a lack of high-risk populations in the early screening programs and a misunderstanding of the condition. With globalization and increased immigration, HbH-disease has gradually achieved recognition as a condition detectable through NBS, particularly in California and some of the Mediterranean and Middle Eastern countries. Over time, the screening laboratory detection methods have improved, and NBS has expanded globally to include populations at higher risk for α-thals. In recent years, screening for Hb H disease has become a priority in some of these countries, particularly in Asia. As an example, since 2014, NBS for Hb H-disease has been the subject of Chinese pilot studies [108] and full population screening in the Philippines [95]. The biggest challenge facing these and other programs screening for Hb Barts is the development of a quantitative cutoff that minimizes the amount of short-term follow-up necessary for maximum case detection [105]. Given the recent report of 2932 cases detected during the first ten years of screening in the Philippines [147], it will be important to review their cutoff-associated molecular data soon to be reported.

## 5. Conclusions

It is clear from the large volume of literature currently addressing NBS for hemoglobinopathies and thalassemias that this is an exciting time, and there is still much left to do. A number of recent publications beyond the scope of this review discuss SCD treatment advances, and references are provided here for the interested reader [203,204,205,206,207,208,209]. Until late 2023, HSCT was the only curative treatment for SCD. At that time, the U.S. Food and Drug Agency approved two one-time gene therapies for treating patients aged 12 years or older with severe SCD [209]. As new treatments are developed that revolutionize survival and quality of life expectations, it is essential that better means of early SCD detection be available globally. 

Readers are encouraged to access the 2023 comprehensive report by the Lancet Haematology Commission [45], which focused on five key areas in defining global strategies for improving SCD outcomes: epidemiology, screening and prevention, treatments/management, therapies with curative potential, and training/education. Perhaps most notable is their conclusion that, “The beginning of the journey would seem to be starting newborn screening programmes in every country with large numbers of people with SCD; although the technology to do this is increasingly available, with POCs and widespread use of mobile phones, it is still a huge undertaking to establish even the most basic programme in most African countries. The end of the journey is also imaginable, in which every patient in the world is offered curative treatment—possibly through the emerging field of in-vivo gene editing, although this requires major technical, logistical, and economic advances, and it would take substantial commitment to make this a reality within the next three decades.” Further, “Although there remains many unknowns concerning the natural history of the disease and uncertainties in relation to the exact burden of sickle cell disease, a series of inexpensive and effective tools to identify people with sickle cell disease, to reduce early mortality, and to prevent severe chronic complications are readily available to be rolled out at scale. There is strong evidence for their benefits and cost-effectiveness, and strategies building on existing infrastructure to maximise investments and effects.”

## Data Availability

No new data were created or analyzed in this study. Data sharing is not applicable to this article.

## Abbreviations

The following abbreviations are used in this manuscript:

CAREST	Caribbean Network of Researchers 705 on Sickle Cell Disease and Thalassemia
CATSA	Comprehensive analysis of thalassemia alleles (China)
CBS	Cord blood specimen
CDC	Centers for Disease Control and Prevention
CE	Capillary electrophoresis
COESCD	Centre of Excellence for Sickle Cell Disease
CONSA	Consortium on Newborn Screening in Africa (Ghana, Kenya, Nigeria, Liberia, Tanzania, Uganda, Zambia)
DBS	Dried bloodspot
DRC	Democratic Republic of the Congo
Hb	Hemoglobin
HPLC	High performance liquid chromatography
HRSA	Health Resources and Services Administration
HSCT	Hematopoietic stem cell transplant
IEF	Isoelectric focusing
LATAM	Latin America
MALDI-TOF	Matrix-assisted laser desorption/ionization time-of-flight
MS/MS	Tandem mass spectrometry
NBS	Newborn bloodspot screening
NCAA	National Collegiate Athletic Association (USA)
NGS	Next-generation sequencing
NHSL	National Hemoglobinopathy Standardization Laboratory (USA)
NSCAEM	National Sickle Cell Anemia Elimination Mission (India)
PEPFAR	President’s Emergency Plan for AIDS Relief (USA)
PKU	Phenylketonuria
POC	Point-of-care
PROPS	Prophylactic Penicillin Study
qPCR	Quantitative polymerase chain reaction
RBSK	Rashtriya Bal Swasthya Karyakram (India)
RUSP	Recommended Uniform Screening Panel
SADaCC	Sickle Africa Data Coordinating Center
SCD	Sickle cell disease
SCD-SC	Hemoglobin SC-Disease
SCD-SS	Hemoglobin SS-Disease (sickle cell anemia)
SCD-Sβ^0^thal	S beta-zero-thalassemia
SCD-Sβ^+^thal	S beta-plus-thalassemia
SCT	Sickle cell trait (HbS carrier)
SMA	Spinal muscular atrophy
SPAN	Sickle Cell Pan African Network
SPARCO	Sickle Pan-African Research Consortium
SSA	Sub-Saharan Africa
WHA	World Health Assembly
WHO	World Health Organization
α-thal	Alpha-thalassemia
β^-^thal	Beta-thalassemia

## References

[1] Guthrie R. (1992). The origin of newborn screening. Screening.

[2] MacCready R.A., Hussey M.G. (1964). Newborn phenylketonuria detection program in Massachusetts. Am. J. Public Health Nations Health.

[3] Alvarez D.A. (1963). Delaware’s phenylketonuria early detection and screening program. Del. Med. J..

[4] Guthrie R., Susi A. (1963). A simple phenylalanine method for detecting phenylketonuria in large populations of newborns. Pediatrics.

[5] Therrell B.L., Adams J. (2007). Newborn screening in North America. J. Inherit. Metab. Dis..

[6] Benson J.M., Therrell B.L. (2010). History and current status of newborn screening for hemoglobinopathies. Semin. Perinatol..

[7] Watson M.S., Mann M.Y., Lloyd Puryear M.A., Rinaldo P., Howell R.R. (2006). Newborn screening: Toward a uniform screening panel and system. Genet. Med..

[8] Murphey W.H., Patchen L., Guthrie R. (1972). Screening tests for argininosuccinic aciduria, orotic aciduria, and other inherited enzyme deficiencies using dried blood specimens. Biochem. Genet..

[9] Schneider R.G., Carter T.P., Willey A.M. (1986). Laboratory identification of hemoglobin variants in the newborn. Genetic Disease Screening and Management.

[10] Garrick M.D., Dembure P., Guthrie R. (1973). Sickle-cell anemia and other hemoglobinopathies. Procedures and strategy for screening employing spots of blood on filter paper as specimens. N. Engl. J. Med..

[11] Schmidt R.M., Brosious E.M., Holland S., Wright J.M., Serjeant G.R. (1976). Use of blood specimens collected on filter paper in screening for abnormal hemoglobins. Clin. Chem..

[12] Garrick M.D. (1989). Newborn screening for sickle cell disease and other hemoglobinopathies. Alternative methods for screening. Pediatrics.

[13] Pearson H.A., O’Brien R.T., McIntosh S., Aspnes G.T., Yang M.M. (1974). Routine screening of umbilical cord blood for sickle cell diseases. JAMA.

[14] Serjeant B.E., Forbes M., Williams L.L., Serjeant G.R. (1974). Screening cord bloods for detection of sickle cell disease in Jamaica. Clin. Chem..

[15] Metters J., Huntsman R.G., Yawson G.I. (1970). The use of the cord blood sample for the detection of sickle-cell anaemia in the newborn. J. Obstet. Gynaecol. Br. Commonw..

[16] Guthrie R. (1969). Screening for phenylketonuria. Triangle.

[17] Congress.Gov. S 2676-National Sickle Cell Anemia Control Act 92nd Congress (1971–1972). https://www.congress.gov/bill/92nd-congress/senate-bill/2676.

[18] Reid C., Rodgers G., Pace B.S. (2007). Sickle cell disease: Demystifying the beginnings. Renaissance of Sickle Cell Disease Research in the Genomic Era.

[19] Pass K.A., Gauvreau A.C., Schedlbauer L., Carter T.P., Carter T.P., Wiley A.M. (1986). Newborn screening for sickle cell disease. Genetic Disease Screening and Management.

[20] El-Haj N., Hoppe C.C. (2018). Newborn screening for SCD in the USA and Canada. Int. J. Neonatal Screen..

[21] Gaston M.H., Verter J.I., Woods G., Pegelow C., Kelleher J., Presbury G., Zarkowsky H., Vichinsky E., Iyer R., Lobel J.S. (1986). Prophylaxis with oral penicillin in children with sickle cell anemia. A randomized trial. N. Engl. J. Med..

[22] Consensus Conference (1987). Newborn screening for sickle cell disease and other hemoglobinopathies. JAMA.

[23] Daniel Y., Elion J., Allaf B., Badens C., Bouva M.J., Brincat I., Cela E., Coppinger C., de Montalembert M., Gulbis B. (2019). Newborn screening for sickle cell disease in Europe. Int. J. Neonatal Screen..

[24] Zeuner D., Ades A.E., Karnon J., Brown J., Dezateux C., Anionwu E.N. (1999). Antenatal and neonatal haemoglobinopathy screening in the UK: Review and economic analysis. Health Technol. Assess..

[25] Streetly A., Latinovic R., Hall K., Henthon J. (2009). Implementation of universal newborn bloodspot screening for sickle cell disease and other clinically significant haemoglobinopathies in England: Screening results for 2005-7. J. Clin. Pathol..

[26] Lobitz S., Telfer P., Cela E., Allaf B., Angastiniotis M., Backman Johansson C., Badens C., Bento C., Bouva M.J., Canatan D. (2018). Newborn screening for sickle cell disease in Europe: Recommendations from a Pan-European Consensus Conference. Br. J. Haematol..

[27] Rahimy M.C., Gangbo A., Ahouignan G., Alihonou E. (2009). Newborn screening for sickle cell disease in the Republic of Benin. J. Clin. Pathol..

[28] Ohene-Frempong K., Oduro J., Tetteh H., Nkrumah F. (2008). Screening newborns for sickle cell disease in Ghana. Pediatrics.

[29] Tshilolo L., Aissi L.M., Lukusa D., Kinsiama C., Wembonyama S., Gulbis B., Vertongen F. (2009). Neonatal screening for sickle cell anaemia in the Democratic Republic of the Congo: Experience from a pioneer project on 31,204 newborns. J. Clin. Pathol..

[30] Knight-Madden J., Lee K., Elana G., Elenga N., Marcheco-Teruel B., Keshi N., Etienne-Julan M., King L., Asnani M., Romana M. (2019). Newborn screening for sickle cell disease in the Caribbean: An update of the present situation and of the disease prevalence. Int. J. Neonatal Screen..

[31] Therrell B.L., Padilla C.D., Borrajo G.J.C., Khneisser I., Schielen P.C.J.I., Knight-Madden J., Malherbe H.L., Kase M. (2024). Current status of newborn bloodspot screening worldwide 2024: A comprehensive review of recent activities (2020–2023). Int. J. Neonatal Screen..

[32] Arjunan A. (2013). A Systematic Evaluation of a Newborn Screening Program for Sickle Cell Disease in Southern Gujarat, India. Master’s Thesis.

[33] World Health Assembly, 59 (2006). Sickle-Cell Anaemia: Report by the Secretariat. World Health Organization. https://iris.who.int/handle/10665/20890.

[34] World Health Assembly, 59 (2006). WHA59.20 Sickle-Cell Anemia. https://apps.who.int/gb/ebwha/pdf_files/WHA59/A59_R20-en.pdf.

[35] World Health Assembly, 63 WHA63/237 Recognition of Sickle-Cell Anaemia as a Public Health Problem. https://digitallibrary.un.org/record/644334/files/A_RES_63_237-EN.pdf.

[36] World Health Assembly, Regional Committee for Africa, 60 (2010). Sickle-Cell Disease: A Strategy for the WHO African Region. AFR/RC60/8. https://apps.who.int/iris/handle/10665/1682.

[37] McCabe E.R., Huang S.Z., Seltzer W.K., Law M.L. (1987). DNA microextraction from dried blood spots on filter paper blotters: Potential applications to newborn screening. Hum. Genet..

[38] Jinks D.C., Minter M., Tarver D.A., Vanderford M., Hejtmancik J.F., McCabe E.R. (1989). Molecular genetic diagnosis of sickle cell disease using dried blood specimens on blotters used for newborn screening. Hum. Genet..

[39] Descartes M., Huang Y., Zhang Y.H., McCabe L.L., Gibbs R., Therrell B.L., McCabe E.R. (1992). Genotypic confirmation from the original dried blood specimens in a neonatal hemoglobinopathy screening program. Pediatr. Res..

[40] Zhang Y.H., McCabe L.L., Wilborn M., Therrell B.L., McCabe E.R. (1994). Application of molecular genetics in public health: Improved follow-up in a neonatal hemoglobinopathy screening program. Biochem. Med. Metab. Biol..

[41] Therrell B.L., Hoppe C., Mann M.Y., Azimi M., Brants A., Brown S.E., Carter L.S., Dorley M.C., Eckman J.R., Flamini M. (2019). Newborn Screening for Hemoglobinopathies.

[42] Hoppe C.C. (2009). Newborn screening for non-sickling hemoglobinopathies. Hematol. Am. Soc. Hematol. Educ. Program.

[43] Al-Awamy B.H., Al-Muzan M., Al-Turki M., Sergeant G.R. (1984). Neonatal screening for sickle cell disease in the Eastern Province of Saudi Arabia. Trans. R. Soc. Trop. Med. Hyg..

[44] Al Arrayed S. (2005). Campaign to control genetic blood diseases in Bahrain. Community Genet..

[45] Piel F.B., Rees D.C., DeBaun M.R., Nnodu O., Ranque B., Thompson A.A., Ware R.E., Abboud M.R., Abraham A., Ambrose E.E. (2023). Defining global strategies to improve outcomes in sickle cell disease: A Lancet Haematology Commission. Lancet Haematol..

[46] Obeagu E.I., John A. (2025). Health equity in sickle cell disease: Overcoming barriers to care in marginalized communities. Ann. Med. Surg..

[47] Obeagu E.I., John A. (2026). Toward a generation free from the burden of sickle cell disease: The role of global partnerships in public health advancement. Pediatr. Blood Cancer.

[48] Minja I.K., Nkya S., Bukini D., Mahenge N., Masamu U., Manongi J., Mgaya J., Mtiiye F., Nkanyemka M., Kisali E.P. (2025). Strengthening global partnerships for sustainable sickle cell disease care: Insights from SickleInAfrica at the 77th United Nations General Assembly and the US-Africa Leaders’ Summit. BMJ Glob. Health.

[49] Shook L.M., Ware R.E. (2026). Advances and Gaps in Global Newborn Screening for Sickle Cell Disease. Int. J. Neonatal Screen..

[50] Mosca A., Paleari R., Palazzi G., Pancaldi A., Iughetti L., Venturelli D., Rolla R., Pavanello E., Ceriotti F., Ammirabile M. (2024). Screening for sickle cell disease: Focus on newborn investigations. Clin. Chem. Lab. Med..

[51] Paleari R., Vidali M., Rolla R., Ceriotti F., Ammirabile M., Mosca A. (2026). Blood collected on dry blood spots is fit for newborn screening of sickle cell disease (SCD) by different analytical systems. Clin. Chim. Acta.

[52] Janda J., Hegert S., Bzdok J., Tesorero R., Holtkamp U., Burggraf S., Schuhmann E., Hörster F., Hoffmann G.F., Janzen N. (2023). High throughput newborn screening for sickle cell disease-application of two-tiered testing with a qPCR-based primary screen. Klin. Padiatr.

[53] Tesorero R., Janda J., Hörster F., Feyh P., Mütze U., Hauke J., Schwarz K., Kunz J.B., Hoffmann G.F., Okun J.G. (2023). A high-throughput newborn screening approach for SCID, SMA, and SCD combining multiplex qPCR and tandem mass spectrometry. PLoS ONE.

[54] El Osta M., Benoist J.F., Naubourg P., Bonacorsi S., Messine R., Ducoroy P., Allaf B. (2024). MALDI-MS in first-line screening of newborns for sickle cell disease: Results from a prospective study in comparison to HPLC. Clin. Chem. Lab. Med..

[55] Renoux C., Roland E., Ruet S., Zouaghi S., Michel M., Joly P., Feray C., Zhao F., Gavanier D., Gaucherand P. (2024). Evaluation of a new tandem mass spectrometry method for sickle cell disease newborn screening. Int. J. Neonatal Screen..

[56] Huang W.X., Cai Y.X., Yang J., Fu S.R., Wang M., Zhang J., Wan K.X., Yu C.W. (2025). Development of a low-cost and high-throughput LC-MS method for newborn screening of thalassemia and abnormal hemoglobin disorders. World J. Pediatr..

[57] Quarmyne M.O., Bock F., Lakshmanan S., Attell B.K., Snyder A., Boudreaux J., Sheth S., Bender M.A., Lal A. (2025). Newborn screening for sickle cell disease and thalassemia. JAMA Health Forum.

[58] McGann T., Hoppe C. (2017). The pressing need for point-of-care diagnostics for sickle cell disease: A review of current and future technologies. Blood Cells Mol. Dis..

[59] Ilyas S., Simonson A.E., Asghar W. (2020). Emerging point-of-care technologies for sickle cell disease diagnostics. Clin. Chim. Acta.

[60] Olaniyan H.S., Briscoe C., Muhongo M., Pascoal R., Armando A., Santos B., McGann P.T. (2023). Early diagnosis of sickle cell disease at birth hospitals and vaccination centers in Angola using point-of-care tests. Blood Adv..

[61] Su J.J., Kupua V.S., Cummings D., Jacobsen K.H. (2025). Newborn screening for sickle cell disease in Caluquembe, southwestern Angola, 2024-2025. PLoS ONE.

[62] Kasai E.T., Kadima J.N., Alworong’a Opara J.P., Boemer F., Dresse M.F., Makani J., Bours V., Marini Djang’eing’a R., Paul K.K., Batina Agasa S. (2022). Pairing parents and offspring’s HemoTypeSC™ test to validate results and confirm sickle cell pedigree: A case study in Kisangani, the Democratic Republic of Congo. Hematology.

[63] Aimé A.K., Etienne S.M., Mbongi D., Nsonso D., Serrao E., Léon T.M.M., Oscar L.N., Stanis W.O. (2022). HemoTypeSC screening for sickle cell disease in the Democratic Republic of Congo (DRC): A case from the city of Kindu. Pan Afr. Med. J..

[64] Mumbere M., Batina-Agasa Uvoya N.A., Kasai E.T., Kombi P.K., Djang’eing’a R.M., Opara J.A. (2023). Newborn screening for sickle cell disease in Butembo and Beni: A pilot experience in a highland region of the Democratic Republic of Congo. Pan Afr. Med. J..

[65] Adegoke S.A., Makalo L., Sallah A., Saine H., Joof S., Conteh A., Bah R., Camara Jammeh A.F., Bass M., Jallow M. (2024). Point-of-care newborn screening for sickle cell disease at selected health facilities in the Gambia. Hemoglobin.

[66] Kakou Danho J.B., Atiméré Y.N., Koné D., Yéo D.D., Couitchéré L. (2021). Feasibility study of the “HemoTypeSC” test for the rapid screening of sickle cell disease in Côte D’Ivoire. Adv. Hematol..

[67] Guindo A., Cisse Z., Keita I., Traore O., Sylla N., Diassana M., Saye A., Picot V., Lauressergues E., Leroy V. (2024). Potential for a large-scale newborn screening strategy for sickle cell disease in Mali: A comparative diagnostic performance study of two rapid diagnostic tests (SickleScan^®^ and HemotypeSC^®^) on cord blood. Br. J. Haematol..

[68] Guindo A., Cablay K., Kamate J., MacLean B., Saye D., Dembele P., Anderson A.R. (2025). Systematic point-of-care newborn screening for sickle cell disease in rural Mali, West Africa. Br. J. Haematol..

[69] Mano R.M., Kuona P., Misihairabgwi J.M. (2024). Determination of birth prevalence of sickle cell disease using point of care test HemotypeSC™ at Rundu Hospital, Namibia. BMC Pediatr..

[70] Oluwole E.O., Adeyemo T.A., Osanyin G.E., Odukoya O.O., Kanki P.J., Afolabi B.B. (2020). Feasibility and acceptability of early infant screening for sickle cell disease in Lagos, Nigeria—Are a pilot study. PLoS ONE.

[71] Christopher H., Josephat E., Kaywanga F., Saul S., Mshana I., Kunambi P., Nasser A., Chamba C., Makani J., Nkya S. (2022). Potential of point of care tests for newborn screening for sickle cell disease: Evaluation of HemotypeSC™ and sickle SCAN^®^ in Tanzania. Int. J. Lab. Hematol..

[72] Purohit P., Parida C., Martha T.K., Bholo S., Naik A., Behera S.K. (2024). Evaluation of a point-of-care rapid diagnostic test kit (SICKLECHECK) for screening of sickle cell diseases. PLoS ONE.

[73] Mukherjee M.B., Colah R.B., Mehta P.R., Shinde N., Jain D., Desai S., Dave K., Italia Y., Raicha B., Serrao E. (2020). Multicenter evaluation of HemoTypeSC as a point-of-care sickle cell disease rapid diagnostic test for newborns and adults across India. Am. J. Clin. Pathol..

[74] Shrivas S., Patel M., Kumar R., Gwal A., Uikey R., Tiwari S.K., Verma A.K., Thota P., Das A., Bharti P.K. (2021). Evaluation of microchip-based point-of-care device “Gazelle” for diagnosis of sickle cell disease in India. Front. Med..

[75] Thaker P., Shinde N., Surve S., Gawit K., Hariharan P., Shanmugam R., Kerketta A.S., Ranjit M., Dave K., Thakor M. (2025). Diagnostic accuracy of HemotypeSC^™^ for detecting sickle cell disease in newborns: A multicentric study. Indian J. Med. Res..

[76] Alvarez O.A., Hustace T., Voltaire M., Mantero A., Liberus U., Saint Fleur R. (2019). Newborn screening for sickle cell disease using point-of-care testing in low-income setting. Pediatrics.

[77] Nnodu O.E., Munung N.S., Chirande L., Chunda-Liyoka C., Kiguli S., Sarfo F.S., Touré B.A., Balandya E., Guindo A., Kuona P. (2024). Acceptability, barriers and facilitators of using dried blood spots-point-of-care testing for sickle cell disease in Africa: An implementation science protocol for a multinational qualitative study. BMJ Open.

[78] Naik R.P., Haywood C. (2015). Sickle cell trait diagnosis: Clinical and social implications. Hematology Am. Soc. Hematol. Educ. Program.

[79] Parker H., Qureshi N., Ulph F., Kai J. (2007). Imparting carrier status results detected by universal newborn screening for sickle cell and cystic fibrosis in England: A qualitative study of current practice and policy challenges. BMC Health Serv. Res..

[80] Hayeems R.Z., Moore Hepburn C., Chakraborty P., Odame I., Clarke J., Miller F.A., Brown A.D. (2017). Managing sickle cell carrier results generated through newborn screening in Ontario: A precedent-setting policy story. Genet. Med..

[81] Lilley M., Hoang S., Blumenschein P., Peturson A.M., Sosova I., Macneil L., Ridsdale R., Christian S. (2021). Sickle cell trait newborn screen results: Disclosure and management. J. Community Genet..

[82] Freppel M., Mention K., Renom G., Lambilliotte A., Barbati M. (2024). Informing parents of newborns with sickle cell trait detected at neonatal screening: A northern France experience. Pediatr. Blood Cancer.

[83] Kavanagh P.L., Wang C., Therrell B.L., Sprinz P.G., Bauchner H. (2008). Communication of positive newborn screening results for sickle cell disease and sickle cell trait: Variation across states. Am. J. Med Genet. C: Semin. Med Genet..

[84] Scott J.L., Christian J., Plazas Montana M., Miller Y.M., Naik R.P. (2024). Variability in notification of positive newborn screening results for sickle cell trait across the United States. Adv. Hematol..

[85] Taber P., Baysinger J., Daniels S., Diaz-Kincaid N., Gaviglio A., Ginter J., Held P.K., Reeves E., Sack V., Weaver J. (2025). Participatory workflow analysis of newborn genetic screening (NBS) to support tools for improved follow-up: Comparing the use case of hemoglobinopathy traits across U.S. States. Int. J. Neonatal Screen..

[86] Tsaras G., Owusu-Ansah A., Boateng F.O., Amoateng-Adjepong Y. (2009). Complications associated with sickle cell trait: A brief narrative review. Am. J. Med..

[87] Anzalone M.L., Green V.S., Buja M., Sanchez L.A., Harrykissoon R.I., Eichner E.R. (2010). Sickle cell trait and fatal rhabdomyolysis in football training: A case study. Med. Sci. Sports Exerc..

[88] Thompson A.A. (2013). Sickle cell trait testing and athletic participation: A solution in search of a problem?. Hematology Am Soc Hematol Educ Program.

[89] Yeargin S.W., Meyer C.M., Hirschhorn R.M., Lane A.D., Arent S.M., Haggard C.R. (2024). Characterization of health and safety resources for athletes with sickle cell trait at NCAA institutions. J. Strength Cond. Res..

[90] Paulukonis S.T., Horiuchi S., Austin T., Vichinsky E.P. (2025). Cohort tracking of sickle cell trait-positive births identified by newborn screening, California, 1991-2013: Public health surveillance for sickle cell trait. Public Health Rep..

[91] Kemper A.R., Knapp A.A., Metterville D.R., Comeau A.M., Green N.S., Perrin J.M. (2011). Weighing the evidence for newborn screening for Hemoglobin H disease. J. Pediatr..

[92] Bender M.A., Yusuf C., Davis T., Dorley M.C., Del Pilar Aguinaga M., Ingram A., Chan M.S., Ubaike J.C., Hassell K., Ojodu J. (2020). Newborn screening practices and alpha-thalassemia detection—United States, 2016. MMWR Morb. Mortal. Wkly. Rep..

[93] Frömmel C. (2018). Newborn screening for sickle cell disease and other hemoglobinopathies: A short review on classical laboratory methods—Isoelectric focusing, HPLC, and capillary electrophoresis. Int. J. Neonatal Screen..

[94] Feuchtbaum L., Carter J., Dowray S., Currier R.J., Lorey F. (2012). Birth prevalence of disorders detectable through newborn screening by race/ethnicity. Genet. Med..

[95] Padilla C.D., Therrell B.L., Alcausin M.M.L.B., de Castro R.C., Gepte M.B.P., Reyes M.E.L., Jomento C.M., Suarez R.C.N., Maceda E.B.G., Abarquez C.G. (2021). Successful implementation of newborn screening for hemoglobin disorders in the Philippines. Int. J. Neonatal Screen..

[96] Therrell B.L., Padilla C.D., Abadingo M.E., Adhikari S.P., Aung T., Aye T.T., Dey S.K., Faizi M., Ganbaatar E., Giang T.T.H. (2025). Consolidated newborn bloodspot screening efforts in developing countries in the Asia Pacific—2024. Int. J. Neonatal Screen..

[97] Charoenkwan P., Taweephol R., Sirichotiyakul S., Tantiprabha W., Sae-Tung R., Suanta S., Sakdasirisathaporn P., Sanguansermsri T. (2010). Cord blood screening for alpha-thalassemia and hemoglobin variants by isoelectric focusing in Northern Thai neonates: Correlation with genotypes and hematologic parameters. Blood Cells Mol. Dis..

[98] Chaibunruang A., Sornkayasit K., Chewasateanchai M., Sanugul P., Fucharoen G., Fucharoen S. (2018). Prevalence of thalassemia among newborns: A re-visited after 20 years of a prevention and control program in Northeast Thailand. Mediterr. J. Hematol. Infect. Dis..

[99] Xie X.M., Zhou J.Y., Li J., Li R., Liao C., Li D.Z. (2015). Implementation of newborn screening for hemoglobin H disease in mainland China. Indian J. Hematol. Blood Transfus..

[100] Mohammed A.M., Al-Hilli F., Nadkarni K.V., Bhagwat G.P., Bapat J.P. (1992). Hemoglobinopathies and glucose-6-phosphate dehydrogenase deficiency in hospital births in Bahrain. Ann. Saudi Med..

[101] Khoriaty E., Halaby R., Berro M., Sweid A., Abbas H.A., Inati A. (2014). Incidence of sickle cell disease and other hemoglobin variants in 10,095 Lebanese neonates. PLoS ONE.

[102] Alkindi S., Al Zadjali S., Al Madhani A., Daar S., Al Haddabi H., Al Abri Q., Gravell D., Berbar T., Pravin S., Pathare A. (2010). Forecasting hemoglobinopathy burden through neonatal screening in Omani neonates. Hemoglobin.

[103] Nasserullah Z., Alshammari A., Abbas M.A., Abu-Khamseen Y., Qadri M., Jafer S.A., Wabel M.A. (2003). Regional experience with newborn screening for sickle cell disease, other hemoglobinopathies and G6PD deficiency. Ann. Saudi Med..

[104] El-Kalla S., Baysal E. (1998). alpha-thalassemia in the United Arab Emirates. Acta Haematol..

[105] Lorey F., Cunningham G., Vichinsky E.P., Lubin B.H., Witkowska H.E., Matsunaga A., Azimi M., Sherwin J., Eastman J., Farina F. (2001). Universal newborn screening for Hb H disease in California. Genet. Test..

[106] Bouva M.J., Sollaino C., Perseu L., Galanello R., Giordano P.C., Harteveld C.L., Cnossen M.H., Schielen P.C., Elvers L.H., Peters M. (2011). Relationship between neonatal screening results by HPLC and the number of α-thalassaemia gene mutations; consequences for the cut-off value. J. Med. Screen..

[107] Goodness S., Cavanagh K., Young W., Shurney W. (2012). Reply: Relationship between neonatal screening results by high-performance, liquid chromatography and the number of alpha-thalassaemia gene mutations: Consequences for the cut-off. J. Med. Screen..

[108] Liao C., Zhou J.Y., Xie X.M., Tang H.S., Li R., Li D.Z. (2014). Newborn screening for Hb H disease by determination of Hb Bart’s using the Sebia capillary electrophoresis system in southern China. Hemoglobin.

[109] Shook L.M., Haygood D., Quinn C.T. (2025). Clinical utility of the addition of molecular genetic testing to newborn screening for hemoglobinopathies for confirmation of alpha-thalassemia trait. Int. J. Neonatal Screen..

[110] Obeagu E.I. (2025). Thalassemia in Sub-Saharan Africa: Epidemiology, diagnosis, and management—A narrative review. Ann. Med. Surg..

[111] Jonani B., Kasule E.C., Herman B.R., Arturo J.F., Mugambwa M.C., Stephen S., Mundaka J.B., Kwizera R., Mboowa G., Bongomin F. (2025). Burden of sickle cell anemia in Africa: A systematic review and meta-analysis. PLoS ONE.

[112] Therrell B.L., Lloyd-Puryear M.A., Ohene-Frempong K., Ware R.E., Padilla C.D., Ambrose E.E., Barkat A., Ghazal H., Kiyaga C., Mvalo T. (2020). Empowering newborn screening programs in African countries through establishment of an international collaborative effort. J. Community Genet..

[113] Satekge T., Okesina A., Anetor J., Erasmus R. (2025). The status of newborn screening in Africa: Situation analysis, future plans and call to action. Afr. J. Lab. Med..

[114] Makani J., Sangeda R.Z., Nnodu O., Nembaware V., Osei-Akoto A., Paintsil V., Balandya E., Kent J., Luzzatto L., Ofori-Acquah S. (2020). SickleInAfrica. Lancet Haematol..

[115] Green N.S., Zapfel A., Nnodu O.E., Franklin P., Tubman V.N., Chirande L., Kiyaga C., Chunda-Liyoka C., Awuonda B., Ohene-Frempong K. (2022). The Consortium on Newborn Screening in Africa for sickle cell disease: Study rationale and methodology. Blood Adv..

[116] Archer N.M., Inusa B., Makani J., Nkya S., Tshilolo L., Tubman V.N., McGann P.T., Ambrose E.E., Henrich N., Spector J. (2022). Enablers and barriers to newborn screening for sickle cell disease in Africa: Results from a qualitative study involving programmes in six countries. BMJ Open.

[117] Egesa W.I., Nakalema G., Waibi W.M., Turyasiima M., Amuje E., Kiconco G., Odoch S., Kumbakulu P.K., Abdirashid S., Asiimwe D. (2022). Sickle cell disease in children and adolescents: A review of the historical, clinical, and public health perspective of Sub-Saharan Africa and beyond. Int. J. Pediatr..

[118] Nnodu O.E., Okeke C.O., Isa H.A. (2024). Newborn screening initiatives for sickle cell disease in Africa. Hematol. Am. Soc. Hematol. Educ. Program.

[119] Nyonator C., Amoah E., Addo E.F., Mukanga M., Asubonteng A.K., Ohene-Frempong K., Spector J.M., Ofori-Acquah S.F. (2023). Access to essential therapy for sickle cell disease in Africa: Experience from a national program in Ghana. Semin. Hematol..

[120] Revvity (2022). PerkinElmer and Novartis Collaborate to Address the Unmet Needs of Sickle Cell Disease in Sub-Saharan Africa. https://news.revvity.com/press-announcements/press-releases/press-release-details/2022/PerkinElmer-and-Novartis-Collaborate-to-Address-the-Unmet-Need-of-Sickle-Cell-Disease-in-Sub-Saharan-Africa/default.aspx.

[121] Zhou A.E., Travassos M.A. (2022). Bringing sickle-cell treatments to children in Sub-Saharan Africa. N. Engl. J. Med..

[122] Dexter D., McGann P.T. (2023). Hydroxyurea for children with sickle cell disease in sub-Saharan Africa: A summary of the evidence, opportunities, and challenges. Pharmacotherapy.

[123] Ally M., Balandya E. (2023). Current challenges and new approaches to implementing optimal management of sickle cell disease in sub-Saharan Africa. Semin. Hematol..

[124] Munung N.S., Nnodu O.E., Moru P.O., Kalu A.A., Impouma B., Treadwell M.J., Wonkam A. (2024). Looking ahead: Ethical and social challenges of somatic gene therapy for sickle cell disease in Africa. Gene Ther..

[125] Odame I., Bazuaye G.N. (2024). Transfusions, disease-modifying treatments, and curative therapies for sickle cell anemia in Africa: Where are we now?. Hematol. Am. Soc. Hematol. Educ. Program.

[126] Mboowa G., Sserwadda I., Kanyerezi S., Tukwasibwe S., Kidenya B. (2025). The dawn of a cure for sickle cell disease through CRISPR-based treatment: A critical test of equity in public health genomics. Ann. Hum. Genet..

[127] Obeagu E.I. (2025). Strategies for reducing child mortality due to sickle cell disease in Uganda: A narrative review. Ann. Med. Surg..

[128] Saidu Y., Masong M.C., Francoise N., Ngenge B.M., Ndansi E., Foma M.K. (2024). Sickle cell disease in Cameroon: Taking out the “neglect” and highlighting key opportunities for sustainable control. PLoS Glob. Public Health.

[129] Nnodu O.E., Sopekan A., Nnebe-Agumadu U., Ohiaeri C., Adeniran A., Shedul G., Isa H.A., Owolabi O., Chianumba R.I., Tanko Y. (2020). Implementing newborn screening for sickle cell disease as part of immunisation programmes in Nigeria: A feasibility study. Lancet Haematol..

[130] Galadanci A.A., Ibrahim U.A., Carroll Y., Jobbi Y.D., Farouk Z.L., Mukaddas A., Hussaini N., Sani Musa B., Klein L.J., DeBaun M.R. (2024). A novel newborn screening program for sickle cell disease in Nigeria. Int. J. Neonatal Screen..

[131] Anie K.A., Treadwell M.J., Grant A.M., Dennis-Antwi J.A., Asafo M.K., Lamptey M.E., Ojodu J., Yusuf C., Otaigbe A., Ohene-Frempong K. (2016). Community engagement to inform the development of a sickle cell counselor training and certification program in Ghana. J. Community Genet..

[132] Mumuni N.D., Osman W., Alhassan B.A., Alhassan A. (2023). Burden experienced by informal caregivers of children with sickle cell disease (SCD): A qualitative exploratory study at Tamale Teaching Hospital, Ghana. BMJ Open.

[133] Ampomah M.O., Atkin K., Ohene L.A., Achempim-Ansong G., Korsah K.A., Laari L. (2024). Financial strain and resilience: A qualitative exploration of parental perspectives on caring for children with sickle cell disease in Ghana. BMC Health Serv. Res..

[134] Queiroz G., Monteiro C., Manco L., Relvas L., Trovoada M.J., Leite A., Bento C. (2024). Sickle cell trait in São Tomé e Príncipe: A population-based prevalence study in women of reproductive age. BMC Public Health.

[135] Freire A., Charola-Ramos L., González-Guerra E., Gonçalves J., Rocha V., Afreixo V., Martínez-Carretero E., Raya J.M. (2024). Sickle cell anemia screening in newborns and analysis of haplotypes in patients from Santiago Island, Cape Verde. Anemia.

[136] Brito M., Catarina Ginete C., Inusa B.P.D., Mendes M., Afonso R., Siatembo A., Vasconcelos J.N. (2024). First-year outcomes of newborn sickle cell disease screening in an Angolan hospital. Blood.

[137] Kasai E.T., Gulbis B., Ntukamunda J.K., Bours V., Batina Agasa S., Marini Djang’eing’a R., Boemer F., Katenga Bosunga G., Ngbonda Dauly N., Sokoni Vutseme J. (2023). Newborn screening for sickle cell disease in Kisangani, Democratic Republic of the Congo: An update. Hematology.

[138] Katamea T., Mukuku O., Mpoy C.W., Mutombo A.K., Luboya O.N., Wembonyama S.O. (2023). Newborn screening for sickle cell disease in Lubumbashi, Democratic Republic of the Congo: An update on the prevalence of the disease. J. Hematol. Allied Sci..

[139] Nangunia N.M., Mukuku O., Feza V.B., Kyembwa Y.M., Kabesha T.B., Tsongo Z.K., Mutombo A.K., Wembonyama S.O. (2025). Integration of newborn screening for sickle cell disease into primary health care in Bukavu, Democratic Republic of the Congo: A pilot study. BMC Prim. Care.

[140] Tegha G., Topazian H.M., Kamthunzi P., Howard T., Tembo Z., Mvalo T., Chome N., Kumwenda W., Mkochi T., Hernandez A. (2021). Prospective newborn screening for sickle cell disease and other inherited blood disorders in central Malawi. Int. J. Public Health.

[141] Orimbo J., Awandu S.S., Muhonja F., Owili P., Omondi D. (2025). High acceptability of newborn screening for sickle cell disease among post-natal mothers in Western Kenya. PLoS ONE.

[142] Ezenwosu O.U., Olawepo J.O., Lacroix-Willliamson L.J., Itanyi I.U., Ogidi A., Onyeka T.C., Gully M., Gregory M., Breeze J.L., Ibemere S. (2024). Health education to promote knowledge about sickle cell disease and newborn screening in pregnant women: A community-based pilot study using the healthy beginning initiative. BMC Pregnancy Childbirth.

[143] Hezekiah A.I., Oparaugo C.I., Ajetomobi G.D., Fasina A.E., Chianumba R.I., Nnodu O.E. (2025). Awareness, attitude, and acceptance of newborn screening for sickle cell disease among health workers and caregivers at primary healthcare centers in Gwagwalada Area Council, Federal Capital Territory, Abuja, Nigeria. Front. Public Health.

[144] Duah E., Chatio S.T., Ababio L.O., Lister N., Egbujo O., Marfo K., Odame I., Ansah P. (2025). The role of counselling to reduce parental fears and anxiety about newborn screening for sickle cell disease in Northern Ghana. BMC Public Health.

[145] Folayan O.S., Orimadegun B.E., Ayede A.I., Inusa B.P., Kase M.K., Anetor J.I. (2026). Integrated newborn screening in Nigeria: The way forward, a workshop report. Int. J. Neonatal Screen..

[146] Kato G.J., Piel F.B., Reid C.D., Gaston M.H., Ohene-Frempong K., Krishnamurti L., Smith W.R., Panepinto J.A., Weatherall D.J., Costa F.F. (2018). Sickle cell disease. Nat. Rev. Dis. Primers.

[147] Chaube P., Singh A.K., Choudhury M.C. (2023). Expansion of India’s national child healthcare programme, Rashtriya Bal Swasthya Karyakram (RBSK), for rare disease management: A health policy perspective. Orphanet J Rare Dis..

[148] Colah R.B., Mehta P., Mukherjee M.B. (2018). Newborn screening for sickle cell disease: Indian experience. Int. J. Neonatal Screen..

[149] Gupta K., Krishnamurti L., Jain D. (2024). Sickle cell disease in India: The journey and hope for the future. Hematol. Am. Soc. Hematol. Educ. Program.

[150] Piel F.B., Colah R., Jain D.L. (2024). Casting light on the national mission to eliminate sickle cell disease in India. Hemasphere.

[151] National Health Mission, Ministry of Health and Family Welfare, Government of India National Sickle Cell Anaemia Elimination Mission—Dashboard. https://sickle.nhm.gov.in/home/guest_dashboard.

[152] National Health Mission Ministry of Health Family Welfare, Government of India National Sickle Cell Anaemia Elimination Mission—Download National, ToT PPT. https://sickle.nhm.gov.in/uploads/user_manual/ppt/SHM_StatExperienc.

[153] Vishnoi A., Jyani R., Sharma A., Poswal L., Goyal S., Swami S.K., Chauhan N.K. (2025). Screening for hemoglobinopathies: An inceptive experience of Centre of Excellence for Sickle Cell Disease. Indian Pediatr..

[154] Rajamani N., Pandey A., Surve S., Desai S., Kulkarni R., Gajbhiye R., Shanmugam R., Dave K., Kerketta A.S., Mohanty S.S. (2025). Barriers, facilitators and recommendations for the implementation of newborn sickle cell screening program in tribal communities: Findings from a qualitative multicentric study in India. Lancet Reg. Health Southeast Asia.

[155] Karra M.L., Rahiman E.A., Narahari M., Bhongir A.V., Rathod G.R.S., Guduru V.K., Kagitapu S., Pyati A., Sampath S. (2025). Unveiling the burden of sickle cell anemia: A pilot study validating dried blood spots for newborn screening. Indian J. Pediatr..

[156] Surve S., Thakor M., Madkaikar M., Kaur H., Desai S., Shanmugam R., Mohanty S.S., Pandey A., Salomi Kerketta A., Dave K. (2025). Protocol for a multicentric cohort study on neonatal screening and early interventions for sickle cell disease among high-prevalence states of India. Diagnostics.

[157] Jilek R., Marcy J., Johnson C., Younger G., Calhoun A., Tung M.L. (2024). Iowa newborn screening program experience with hemoglobinopathy screening over the last two decades and its increasing global relevance. Int. J. Neonatal Screen..

[158] Kayle M., Blewer A.L., Pan W., Rothman J.A., Polick C.S., Rivenbark J., Fisher E., Reyes C., Strouse J.J., Weeks S. (2024). Birth prevalence of sickle cell disease and county-level social vulnerability—Sickle cell data collection program, 11 states, 2016–2020. MMWR Morb. Mortal. Wkly. Rep..

[159] Therrell B.L., Lloyd-Puryear M.A., Eckman J.R., Mann M.Y. (2015). Newborn screening for sickle cell diseases in the United States: A review of data spanning 2 decades. Semin. Perinatol..

[160] Miller J.I., Hassell K.L., Kellar-Guenther Y., Quesada S., West R., Sontag M. (2024). The prevalence of sickle cell disease in Colorado and methodologies of the Colorado sickle cell data collection program: Public health surveillance study. JMIR Public Health Surveill..

[161] Hays L., DuBois D., Saccente S., Schaefer G. (2024). Clinical treatment patterns and outcomes of sickle cell from the Arkansas newborn screening long term follow-up database study: 2011–2023. Gen. Med. Open.

[162] Kazadi C., Ducruet T., Forté S., Robitaille N., Pastore Y. (2024). Positive impacts of universal newborn screening on the outcome of children with sickle cell disease in the province of Quebec: A retrospective cohort study. eJHaem.

[163] Volodko N., Parker M.L., Ridsdale R., MacNeil L., Sosova I., Estey M.P., Proctor D., Olayinka L., Newbigging A., Bordeleau P. (2026). Hemoglobin Chapel Hill masquerading as hemoglobin S in newborn sickle cell screening: A case study. Clin. Biochem..

[164] Bain B.J., Daniel Y., Henthorn J., de la Salle B., Hogan A., Roy N.B.A., Mooney C., Langabeer L., Rees D.C., B.S.H. Committee (2023). Significant haemoglobinopathies: A guideline for screening and diagnosis: A British Society for Haematology Guideline. Br. J. Haematol..

[165] Marco Sánchez J.M., Bardón Cancho E.J., Benéitez D., Payán-Pernía S., Collado Gimbert A., Ruiz-Llobet A., Salinas J.A., Sebastián E., Argilés B., Bermúdez M. (2024). Haemoglobinopathies and other rare anemias in Spain: Ten years of a nationwide registry (REHem-AR). Ann. Hematol..

[166] Sánchez-Villalobos M., Campos Baños E., Juan Fita M.J., Egea Mellado J.M., Gonzalez Gallego I., Beltrán Videla A., Berenguer Piqueras M., Bermúdez Cortés M., Moraleda Jiménez J.M., Guillen Navarro E. (2023). A newborn screening program for sickle cell disease in Murcia (Spain). Int. J. Neonatal Screen..

[167] González de Aledo-Castillo J.M., Argudo-Ramírez A., Beneitez-Pastor D., Collado-Gimbert A., Almazán Castro F., Roig-Bosch S., Andrés-Masó A., Ruiz-Llobet A., Pedrals-Portabella G., Medina-Santamaria D. (2024). Newborn screening for sickle cell disease in Catalonia between 2015 and 2022—Epidemiology and impact on clinical events. Int. J. Neonatal Screen.

[168] Núñez-Jurado D., Payán-Pernía S., Álvarez-Ríos A.I., Jiménez-Jambrina M., Concepcion Pérez-De-Soto I., José Palma-Vallellano A., Zapata-Bautista R., Carlos Hernández-Castellet J., Paz Garrastazul-Sánchez M., Arqueros-Martínez V. (2024). Neonatal screening for sickle cell disease in Western Andalusia: Results and lessons learnt after 3 years of implementation. Am. J. Perinatol..

[169] García-Morin M., Bardón-Cancho E.J., Beléndez C., Dulín E., Blanco-Soto P., Puertas-López C., Prieto-Medina M., Cervera-Bravo Á., Llorente-Otones L., Pérez-Alonso V. (2024). Madrid newborn sickle cell disease cohort: Clinical outcomes, stroke prevention and survival. Ann. Hematol..

[170] Bzdok J., Czibere L., Burggraf S., Pauly N., Maier E.M., Röschinger W., Becker M., Durner J. (2024). A modular genetic approach to newborn screening from spinal muscular atrophy to sickle cell disease-Results from six years of genetic newborn screening. Genes.

[171] Testa E.R., Robazza M., Barbieri F., Travan L., Miani M.P., Miorin E., Toller I., Dragovic D., Moretti V., Facchin S. (2024). Ten years of a neonatal screening program for hemoglobinopathies in Friuli-Venezia Giulia: First regional experience in Italy. Blood Transfus..

[172] Vuong C., Eckhardt C.L., Heijboer H., Suijker M.H., de Ligt L.A., Voigt A.L.A., Leeflang M.M.G., Bartels M., Brons P., Hooimeijer L. (2024). Long-term follow-up of children with sickle cell disease diagnosed by newborn screening in the Netherlands: Overview of morbidity and mortality. Am. J. Hematol..

[173] Rodrigues D., Marcão A., Lopes L., Ventura A., Faria T., Ferrão A., Gonçalves C., Kjöllerström P., Castro A., Fraga S. (2025). Newborn screening for sickle cell disease: Results from a pilot study in the Portuguese population. Int. J. Neonatal Screen..

[174] Service-Public.Fr (2024). Sickle Cell Disease Screening for Newborns from November 1 [2024]. https://www.service-public.fr/particuliers/actualites/A17573.

[175] Long J., Yu C., Sun L., Peng M., Song C., Mao A., Zhan J., Liu E. (2024). Comprehensive analysis of thalassemia alleles (CATSA) based on third-generation sequencing is a comprehensive and accurate approach for neonatal thalassemia screening. Clin. Chim. Acta.

[176] Li H., Wei J., Zhao H. (2024). Analysis of hemoglobin electrophoresis results and their clinical significance in neonates from Beiliu City. Adv. Biosci. Biotechnol..

[177] Su Y., Xie J., He J., Shen Y., Li T., Huang W., Tong X., Bian Q. (2025). Screening and treatment of thalassemia. Clin. Chim. Acta.

[178] Tang M.J., Roosblad J., Codrington J., Peters M., Toekoen A., van Rheenen P., Juliana A. (2024). Evaluation of the newborn screening pilot for sickle cell disease in Suriname using the non-adoption, abandonment, scale-up, spread, and sustainability (NASSS) framework. Int. J. Neonatal Screen..

[179] Elenga N., Ro V., Mafema Missindu J., Thomas Boizan N., Vaz T., Lucarelli A., Armoudon-Fleret M.É., Buendé S. (2024). Sickle cell disease in French Guiana: Assessing 30 years of neonatal screening (1992-2021)]. Med. Trop. Sante Int..

[180] Saint Fleur R., Archer N., Hustace T., Louis R.J., Bellevue R., Gautier J., Lerebours E., Voltaire M., Alvarez O. (2018). Capacity building and networking to make newborn screening for sickle cell disease a reality in Haiti. Blood Adv..

[181] Alvarez O.A., St Victor Dély N., Paul Hanna M., Saint Fleur R., Cetoute M., Metalonis S., Hustace T., Brown E.C., Marcelin L.H., Muscadin E. (2024). Implementation of hospital-based sickle cell newborn screening and follow-up programs in Haiti. Blood Adv..

[182] Belle Jarvis S., Hadeed E., Lee K., Hardy-Dessources M.D., Knight-Madden J.M., Richardson C. (2023). Sickle cell disease newborn screening—An audit of a twin island state pilot program. Int. J. Neonatal Screen..

[183] Silva-Pinto A.C., Alencar de Queiroz M.C., Antoniazzo Zamaro P.J., Arruda M., Pimentel Dos Santos H. (2019). The neonatal screening program in Brazil, focus on sickle cell disease (SCD). Int. J. Neonatal Screen..

[184] Mota C.S., Lira A.D.S., Queiroz M.C.A., Santos M.P.A.D. (2024). Àgô Sankofa: An overview of the progression of sickle cell disease in Brazil in the past two decades. Cien Saude Colet..

[185] Costa R.D., Ferreira M.F.C., Rocha T.A., Galera M.F. (2023). Evaluation of newborn screening in the state of Mato Grosso from 2005 to 2019. Rev. Paul. Pediatr..

[186] Albanés E.J., Jacinto A.C., Chiroy A.S., Gaitán I.C., Tzorín P.A. (2022). Preliminary incidence of structural hemoglobinopathies in Guatemala. J. Inborn Errors Metab. Screen..

[187] Bernal J.E., Tamayo M.L., Briceño I., Benavides E. (2024). Newborn screening in Colombia: The experience of a private program in Bogotá. Biomedica.

[188] Chaouch L., Moumni I., Ben Abdallah J., Bouchahda R., Methlouthi J., Mahdhaoui N., Matamri W., Braham N., Bouguila F., Mejri L. (2024). Newborn screening of hemoglobinopathies in a center Tunisian population. J. Pediatr. Hematol. Oncol..

[189] Ouragini H., Halim N.B., Zitouni S., Chaouachi D., Boudrigua I., Saidani N., Kraiem I., Ayachi A., Abbes S., Mourali M. (2025). Cord blood-based neonatal screening for hemoglobinopathies in Northern Tunisia. Int. J. Neonatal Screen..

[190] El Janahi S., Filali M., Boudar Z., Akhattab A., El Jaoudi R., Al Idrissi N., Dini N., Nejjari C., Yahyaoui R., Lloyd-Puryear M.A. (2024). Newborn screening for six primary conditions in a clinical setting in Morocco. Int. J. Neonatal Screen..

[191] Ezzat H., Alshehri M., Alghamdi I., Alshehri F., Kariri N., Albalawi F., Aldoaiji N., Al Abdulaali M. (2024). Newborn screening program (NBS) for early detection of sickle cell disease and G6PD: The Saudi Experience. Blood.

[192] United Nations. Department of Economic and Social Affairs Transforming Our World: The 2030 Agenda for Sustainable Development. https://sdgs.un.org/2030agenda.

[193] World Health Assembly, 77. WHA77.5. Accelerating Progress Towards Reducing Maternal, Newborn and Child Mortality in Order to Achieve Sustainable Development Goal Targets 3.1 and 3.2. 1 June 2024. https://apps.who.int/gb/ebwha/pdf_files/WHA77/A77_R5-en.pdf.

[194] Iroh Tam P.Y., Padilla C.D., Zlotkin S., Ayede A.I., Banu T., Kayita J., Khanna R., Rao S.P., Siddeeg K., Walani S. (2024). The 77th World Health Assembly resolution calling for newborn screening, diagnosis, and management of birth defects: Moving towards action in low-income and middle-income countries. Lancet Glob. Health.

[195] Shook L.M., Haygood D., Quinn C.T. (2021). Clinical utility of the addition of molecular genetic testing to newborn screening for sickle cell anemia. Front. Med..

[196] Galadanci N.A., Ibrahim U.A., Ilonze C. (2026). Newborn screening for sickle cell disease in the United States: Gaps in medical care for the pediatric clinicians. Pediatr. Clin. N. Am..

[197] Okeke C.O., Okeke C., Asala S., Ofakunrin A.O.D., Ufelle S., Nnodu O.E. (2024). Sustainability of newborn screening for sickle cell disease in resource-poor countries: A systematic review. PLoS ONE.

[198] Kuznik A., Habib A.G., Munube D., Lamorde M. (2016). Newborn screening and prophylactic interventions for sickle cell disease in 47 countries in sub-Saharan Africa: A cost-effectiveness analysis. BMC Health Serv. Res..

[199] Grosse S.D., Odame I., Atrash H.K., Amendah D.D., Piel F.B., Williams T.N. (2011). Sickle cell disease in Africa: A neglected cause of early childhood mortality. Am. J. Prev. Med..

[200] Knight-Madden J., Romana M., Villaescusa R., Reid M., Etienne-Julan M., Boutin L., Elana G., Elenga N., Wheeler G., Lee K. (2016). CAREST--Multilingual Regional Integration for Health Promotion and Research on Sickle Cell Disease and Thalassemia. Am. J. Public Health.

[201] Serjeant B.E., Mason K., Reid M., Hambleton I., Serjeant G.R. (2024). The aplastic crisis in HbSS: Observations from the Jamaican birth cohort. Hemoglobin.

[202] Serjeant G., Mason K., Hambleton I., Serjeant B. (2024). Acute splenic sequestration in HbSS: Observations from the Jamaican birth cohort. Arch. Dis. Child..

[203] Dimitrievska M., Bansal D., Vitale M., Strouboulis J., Miccio A., Nicolaides K.H., El Hoss S., Shangaris P., Jacków-Malinowska J. (2024). Revolutionising healing: Gene editing’s breakthrough against sickle cell disease. Blood Rev..

[204] Butt H., Sathish S., London E., Lee Johnson T., Essawi K., Leonard A., Tisdale J.F., Demirci S. (2025). Genome editing strategies for targeted correction of β-globin mutation in sickle cell disease: From bench to bedside. Mol. Ther..

[205] Ahmed R., Alghamdi W.N., Alharbi F.R., Alatawi H.D., Alenezi K.M., Alanazi T.F., Elsherbiny N.M. (2026). CRISPR/Cas9 System as a promising therapy in thalassemia and sickle cell disease: A systematic review of clinical trials. Mol. Biotechnol..

[206] Almasoudi H.H. (2025). Therapeutic promise of CRISPR-Cas9 gene editing in sickle cell disease and β-thalassemia: A current review. Curr. Res. Transl. Med..

[207] Ball J., Bradley A., Le A., Tisdale J.F., Uchida N. (2025). Current and future treatments for sickle cell disease: From hematopoietic stem cell transplantation to in vivo gene therapy. Mol. Ther..

[208] Ball J., Bradley A., Le A., Tisdale J.F., Uchida N. (2025). Hematopoietic stem cell therapy with gene modification to treat sickle cell disease. Stem Cells Transl. Med..

[209] Kansal R. (2025). Curing sickle cell disease by allogeneic hematopoietic stem cell (HSC) transplantation toward in vivo HSC gene therapy. Genes.

